# Breaking down malaria outbreak: A multidisciplinary approach in a border village of French Guiana

**DOI:** 10.1371/journal.pntd.0013096

**Published:** 2025-06-17

**Authors:** Hélène Tréhard, Guillaume Lacour, Emilie Mosnier, Amandine Guidez, Yanouk Epelboin, Yassamine Lazrek, Felix Djossou, Jean Gaudart, Isabelle Dusfour, Lise Musset

**Affiliations:** 1 UMR1252 SESSTIM, Aix-Marseille Univ, Inserm, IRD, Aix Marseille Institute of Public Health ISSPAM, Marseille, France; 2 Vectopôle Amazonien Emile Abonnenc, Institut Pasteur de la Guyane, Cayenne, Guyane, France; 3 Altopictus, Pérols, France; 4 Grant Management Office, University of Health Sciences, Phnom Penh, Cambodia; French Agency for Research on AIDS, Viral Hepatitis and Emerging Infectious diseases (ANRS-MIE), Phnom Penh, Cambodia,; 5 Laboratoire de parasitologie, World Health Organization Collaborating Center for Surveillance of Antimalarial Drug Resistance, Centre National de Référence du Paludisme, Institut Pasteur de la Guyane, Cayenne, Guyane, France; 6 Unité des Maladies Infectieuses et Tropicales, Centre Hospitalier de Cayenne, French Guiana, France; 7 Aix Marseille Univ, INSERM, IRD, ISSPAM, SESSTIM, UMR1252, APHM, Hop Timone, BioSTIC, Biostatistic & ICT, Marseille, France; Institut Pasteur, FRANCE

## Abstract

**Background:**

Isolated areas of malaria transmission can persist in countries nearing malaria elimination. To tailor interventions accordingly, smaller-scale surveillance and a deeper understanding of local conditions are needed, including human, environmental and vectorial parameters, and how these parameters interact. This study investigated the potential at-risk situations that could explain the dynamics of malaria persistence in an isolated recurrent high transmission area in French Guiana, using a transdisciplinary approach.

**Methodology/Principal Findings:**

We implemented an ancillary study in Trois-Palétuviers (200 inhabitants), an isolated village in French Guiana bordering Brazil. This was based on two cross-sectional surveys conducted from September to December in 2017 and 2018 that collected both malaria prevalence and behavioural data. Entomological data were collected using 4 Mosquito Magnet traps for three consecutive nights each month. A total of 182 participants were included. The median age was 12.5 years in 2017 (IQR [5;27]). Malaria PCR prevalence was 51% (n = 92) in 2017 and 16% (n = 30) in 2018. Almost all participants used bed nets (99%, n = 180) – but 88% had at least one factor of bed nets ineffectiveness (mainly inadequate drying and washing) –, 63% (n = 114) used indoor insecticides and 32% (n = 57) used skin repellents. *Anopheles darlingi*, representing 99.7% of the *Anopheles* caught, were captured throughout the night, with higher abundance in the evening and early morning. No *An. darlingi* were found in slash-and-burn fields. The high relative abundance of *An. darlingi* before bedtime exposed inhabitants to the vector’s bites.

**Conclusions/Significance:**

The absence of *An. darlingi* in expected locations, such as slash-and-burn fields, points to the complex ecology of malaria transmission and underscores the need for a nuanced understanding of environmental influences, with continuous surveillance. Cross-description of the data suggests a high risk of exposure to mosquito bites by residents before they are protected by bed nets. The inadequacy of anti-vectorial protective measures could be mitigated by education campaigns about protective tools, and by additional protective tools and a frequent distribution of bed nets.

## Author summary

In areas nearing malaria elimination, where only a few cases remain, these are either sporadically disseminated throughout the country or found in hotspots where epidemics are recurrent. These persisting pockets need to be thoroughly investigated to understand the human, entomological, and environmental conditions that explain their persistence.

Our work focuses on one of these areas in French Guiana, where two micro-epidemics occurred in 2017 and 2018 in a border village of 200-inhabitants. We described the location and nightly dynamics of the main malaria vector in the area, *Anopheles darlingi*, as well as the potential at-risk and protective human behaviours when and where that vector is present. The inadequacy of anti-vectorial protection for outdoor and evening activities, despite the presence of the vector, highlights the need to supplement bed nets with other acceptable and free protection. New acceptable protection needs to be developed against outdoor bites, with complementary strategies added in the meantime. The persistence of malaria vectors at night indicates that frequent bed net distribution should be maintained, along with education about bed net care. Additionally, education campaigns about protective tools and prompt healthcare-seeking are also required.

## Introduction

Malaria remains a major global disease, with 608,000 deaths reported in 2022, primarily in Africa [1]. However, due to intensive malaria control interventions and favourable health policies, many countries have succeeded in eliminating or significantly reducing their number of cases [1]. In some of these countries nearing elimination, isolated pockets of malaria transmission persist in specific areas despite ongoing efforts to fight the remaining cases [1,2]. To address malaria transmission in these final phases of elimination, the World Health Organization recommends adopting smaller-scale surveillance and gaining a deeper understanding of local conditions to tailor interventions accordingly [3]. In line with this approach, understanding local malaria epidemics and identifying factors responsible for the persistence of cases in these pockets are crucial to adapt interventions at a local level. Previous studies of these persistent pockets have identified factors contributing to the persistence of malaria such as limited access to healthcare, specific exposures, lack of protective measures, border mobility, and asymptomatic reservoirs [4,5].

For such situations, a good comprehension of the malaria reservoir as well as possible at-risk exposures are essential. An epidemiological description of cases and an overview of available protective tools are essential to better understand the risks of exposure, but studying behaviour that could either expose individuals to the vector or protect them from it is also crucial (...). The local dynamics of vector activity are also a major source of spatio-temporal disparities in the risk of malaria transmission and must therefore be understood to obtain a complete picture of the situation [6]. Finally, these various data need to be cross-analyzed to specifically examine at-risk situations of human-vector interactions [7]. While this concept is longstanding, its field implementation is challenging as it requires the integration of different disciplines and suitable financing [8]. This complexity may explain why, despite its importance for understanding situations in these pockets, interdisciplinary research on vector-borne diseases like malaria remains insufficient [9].

French Guiana is one of the areas nearing malaria elimination, with remaining pockets of transmission in the forest (associated with gold mining activities) and along its border with Brazil. This persistence is suspected to be linked to the presence of the vector, cross-border mobility, and traditional agriculture, as highlighted in previous studies [10–13]. Despite a significant decrease in cases since 2010, two local epidemics occurred in 2017 and 2018, particularly affecting Saint-Georges (SGO), the main French town on the border with Brazil [14]. Asymptomatic cases have been shown to be a potential reservoir and factors such as slash-and-burn farming and travelling to the neighbouring Brazilian indigenous territories have been linked to *Plasmodium* spp. carriage in previous studies [14]. Trois-Palétuviers was the most affected village in the neighbourhoods of this town [14].

Our study objective was to determine the potential situations (risks of exposure and absence of protection in a specific environment) that could expose inhabitants to malaria and explain the high transmission rate and persistence of malaria in Trois-Palétuviers, using a transdisciplinary approach integrating human, vectorial, and environmental factors.

## Methods

### Ethics statements

The PALUSTOP study was approved by the Comité de Protection des Personnes du Sud-Ouest et Outre-Mer 4 N° AM-36/1/CPP15–024. Prior to the study, written informed consent was obtained from all participants. Formal written consent was obtained from the parent/guardian for child participants. The database of the PALUSTOP prospective study was anonymized and declared to the French data protection commission (Commission Nationale Informatique et Libertés, CNIL, n°917186).

### Study site

Trois-Palétuviers is a remote neighbourhood of SGO located on the border between French Guiana and Brazil, along the Oyapock river. It can only be accessed by a 1-hour boat trip from the city centre where the health centre is located. It is surrounded by the Amazonian forest. Trois-Palétuviers has approximately 200 inhabitants out of 4,245 inhabitants in the town (according to health centre data and 2019 census) [15]. The only public services available in Trois-Palétuviers are a primary school and an outpost clinic of the government-funded SGO health centre, where inhabitants can visit a nurse and/or a doctor once a week. The neighbourhood is expanding by reclaiming land from the forest. Housing varies from informal structures to brick houses. Electricity is provided by a petrol generator, and potable water is supplied by two boreholes. Middle school students commute to school via the 1-hour boat trip to SGO city centre every day. The nearest referral hospital is located in Cayenne, a 2 and a half hours’ drive from the SGO city centre. The nearest Brazilian hospital is located in Oiapoque, the main town on the border, 15 minutes by boat from the SGO city centre, and 1 hour by boat from Trois-Palétuviers. Malaria detection and treatment are also available in Taparabu, on the Brazilian side of the river, 15 minutes by boat from Trois-Palétuviers, though with occasional supply issues.

Malaria control interventions in Trois-Palétuviers around the study period included passive case detection in the health centre (Rapid Diagnosis Test -RDT- for all fevers without other apparent causes), sporadic free distribution of impregnated bed nets in 2017, outdoor ultra-low volume spraying (Aqua K-Othrine) and indoor residual spraying on walls (when building types were compatible) on the 14^th^ September 2017, and treatment of neighbouring marshy areas with larvicide (*Bacillus thuringiensis israelensis*) in September 2017, May 2018 and September 2018. All these interventions were performed by government health authorities [14].

No vector collections had been conducted in this specific neighbourhood before our study, but the main vector in French Guiana and in other SGO neighbourhoods is *Anopheles darlingi* [16]. *Anopheles darlingi* is mostly active at night, with high numbers observed in the early evening and before sunset. It bites both indoor and outdoor, targeting mainly humans but also animals like dogs, goats, pigs and cows [17].

The climate of the area is equatorial, hot and wet with a constant monthly mean temperature around 27 °C. The mean annual rainfall is 3,345 mm, with monthly mean amounts ranging from a minimum of 40 mm in September to a maximum of 533 mm in May. There are four alternating seasons: a long rainy season from April to June; a long dry season from July to December; a short rainy season from January to February; and a short dry season in March [16,18].

### Study design

We designed an ancillary study combining data from the PALUSTOP program implemented in SGO from September 2017 to December 2018 [19] and an entomological investigation implemented at the end of 2017 and during 2018, in reaction to the 2017 epidemic. Our study focusses on the data collected in the Trois-Palétuviers neighbourhood, the most affected neighbourhood during the 2017 epidemics. Of the 200 inhabitants, 183 were included in the PALUSTOP active case detection and knowledge study about malaria. For our ancillary study, we chose to exclude an outlier with a specific behaviour regarding mosquito protection compared to the local population.

The PALUSTOP study aimed to better understand the epidemics in SGO through an integrative approach, including two surveys for human biological and behavioural data collection (i.e., September to December 2017 and September to December 2018), as well as six entomological data collections (i.e., September, October and November 2017 and August, September and October 2018) (S1 Fig). The two surveys for human biological and behavioural data collection also included a test and treat strategy.

All children and adults were invited to participate. Those who agreed underwent malaria testing both years using a Polymerase Chain reaction (PCR) test. Latitude and longitude coordinates of participants’ homes were collected. Blood samples were collected by nurses, and questionnaires were administered by trained community health workers (CHW) to limit any potential misunderstanding related to cultural and language barriers. All persons who tested positive for PCR, received treatment following regional guidelines, whether symptomatic or not [20]. *Plasmodium falciparum* infections were treated with artemether-lumefantrine and *P. vivax* infections with chloroquine for 3 days (25mg/kg) followed by 14 days of primaquine (0.5mg/kg) in the absence of medical contraindications.

Analysis focusing on asymptomatic reservoir assessment, malaria knowledge and factors associated with malaria at SGO scale has already been published. The potential at-risk situations combining risks of exposure (studying vector localisation and nocturnal human outdoor activities), and lack of protection had not been investigated until now.

### Variables and data sources

#### Sociodemographic data.

The age, sex, number of people in the household, number of years spent in Trois-Palétuviers, nationality and ethnic group of participants were collected to provide an overview of the sociodemographic structure of the area. Age, sex and ethnic group have been correlated with *Plasmodium* spp. carriage in the area (malaria carriage was found to be higher in young men and in Amerindian villages) [19,21]. Ethnic groups were categorized into Amerindians (i.e., speak at least one Amerindian language as their mother tongue) and others (i.e., no Amerindian language spoken as their mother tongue).

#### Access to care and healthcare pathway.

Data on the social security system and healthcare behaviours (i.e., the health centre visited during their last fever episode) were collected to assess the healthcare pathway and access to care.

#### Malaria knowledge.

Three questions were used to assess malaria knowledge: 1) Have you ever heard about malaria? 2) What do you think transmits malaria? (Options included: air, touching someone with malaria, drinking water, food, mosquitoes, other, don’t know) 3) Is there any way to avoid catching malaria? (Options included: bed nets, skin repellents, indoor insecticides, long clothing, medication, plants, fan, emptying water bowls, spraying in the village (street crossing), other, none, don’t know). Participants who mentioned at least mosquitoes as a mode of malaria transmission were considered to have correct knowledge. These knowledge questions focused on participants aged 7 and over.

#### Risk of exposure.

The potential risks of exposure included practising activities outdoors at night (from sunset to sunrise) and/or in high-risk areas, as reported by participants during questionnaire administration. This included exposures outside the neighbourhood, such as travelling to high-risk areas, visits to gold mining sites, and outdoor activities for subsistence, work, or recreation (slash-and-burn farming, hunting, fishing). Participants reporting at least one visit to a gold mining site were considered potentially exposed. Travel to high-risk areas was defined as reporting at least one trip to the Oyapock Indigenous Territories (OIT), located on the other side of the Oyapock river in Brazil, which has historical connections with Trois-Palétuviers and probable links to malaria in SGO [14,22]. For outdoor activities, participants were considered potentially exposed if they reported frequent (more than three times a week) or occasional fishing, slash-and-burn farming, or hunting, and if they mentioned doing it at night. Additionally, participants who reported going to slash-and-burn fields before 7 AM and/or returning at 6 PM or later were considered potentially exposed based on the entomological findings.

Regarding exposures within the village, data were collected on waking times, playing football after 6 PM, and watching TV without bed nets, reflecting our understanding of the inhabitants’ lifestyle. Waking up before 7 AM was considered potential exposure based on the entomological results. No specific information was collected regarding participants’ TV viewing schedules, so it was assumed they watched TV until bedtime. Lastly, based on the entomological results, proximity to the forest was considered potentially at-risk and was calculated as the distance between participants’ homes (GPS coordinates) and the nearest forest edge, using aerial photography from the French government’s web mapping service (Geoportail) taken on August 30^th^ 2018.

#### *Plasmodium* spp. infection.

Two types of data on *Plasmodium* spp. infection were collected annually. The first type included participants with at least one *Plasmodium* spp. episode diagnosed by RDT at the SGO health centre in 2017 and/or 2018, based on passive data collection from health records (including weekly diagnoses at the Trois-Palétuviers outpost clinic). Episodes occurring within 90 days after a documented episode were considered relapses and were excluded from our survey (n = 48 relapses) [23]. The RDT test used was the SD BIOLINE Malaria Ag Pf/Pan test (pfHRP2/pLDH-based, Standard Diagnostics).

The second type of data consisted of positive cases identified by PCR (using venous blood) during the PALUSTOP 2017 and/or 2018 surveys. Detection and identification of four *Plasmodium* species (*P. falciparum, P. vivax, P. ovale, and P. malariae*) were performed using a Taqman (Vilnius, Lithuania) probe strategy, with a sensitivity of 1 parasite/μL for all the species except *P. vivax* at 0.25p/μL [24].

In our study, a malaria carrier was defined as having had at least one *Plasmodium* spp. episode diagnosed by RDT at the SGO health centre in 2017 and/or 2018, and/or at least one positive PCR result in the PALUSTOP 2017 and/or 2018 surveys.

#### Protection used against mosquitoes.

Information was collected regarding the use of bed nets, windows screen, skin repellent and indoor insecticide (spray and/or coil). Participants were considered to be using a protection if they reported using skin repellent or indoor insecticide often (i.e., every day or almost every day) or sometimes.

Further exploration of bed net use included additional questions for those who reported using them: Are the bed nets impregnated with insecticides? Are there holes in the bed nets? When did you get your bed nets (Less than a year ago, between 1 and 2 years ago, between 2 and 3 years ago, between 3 and 4 years ago, more than 4 years ago, don’t know)? How do you wash your bed nets (washing machine, by hand, don’t wash)? How often do you wash your bednets (Once a week, once a month, once a year, never, other)? Do you dry your bed nets in the sun after washing them?

We created a score based on the sum of potential factors of ineffectiveness (equally weighted) collected through the questionnaire. These ineffectiveness factors were defined as follows: bed nets not impregnated with insecticides, bed nets with holes, bed nets over 2 years old, inadequate washing (using a washing machine or not washing them), inadequate washing frequency (more or less than once a month) and sun drying. These ineffectiveness factors were based on literature suggesting that impregnated bed nets lose their effectiveness with time, as well as with frequent washing, with the use of washing machines and sun drying [25]. Not washing the bed nets also decreases their effectiveness due to the accumulation of dust [25,26].

#### Entomological data.

**Fig 1:**
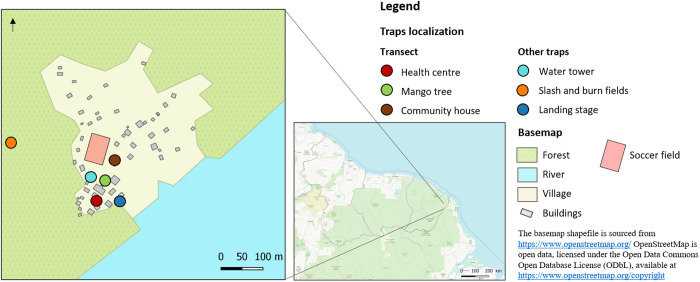
Participants and trap localisation. The basemap shapefile is sourced from https://www.openstreetmap.org/ OpenStreetMap is open data, licensed under the Open Data Commons Open Database License (ODbL), available at https://www.openstreetmap.org/copyright.

To collect mosquitoes, 2–4 Mosquito Magnet (MM) traps were deployed for at least 3 consecutive nights per month, 3 months per year (weeks 37-41-45 in 2017 and weeks 34-37-40 in 2018) (Figs 1 and S1). Three MM were arranged to form a transect through the neighbourhood, extending from the forest to the neighbourhood centre. The MM traps were set outside in an open space but less than 20 metres from a potential resting site: the health centre, a single Mango tree, and the community carbet, a traditional wooden shelter without walls. The trap located near the health centre was set up every night during each survey period, while the others were set up alternately (Table 1). Additional MM traps were placed in a slash-and-burn field within the forest near the neighbourhood, on the landing stage near the river, and at the base of the water tower near the forest (Fig 1). The MM traps were set up in the south of the village to limit the risk of the pack of wild dogs attacking the agents when the MM nets were changed at night, and to limit their barking for the villagers trying to sleep. From August to October, MM traps were operational from 6 PM to 7 AM in 2017 and from 5 PM to 8 AM in 2018, with hourly collections except from midnight to 5 AM, during which only one collection was made at 3 AM. In November 2017, MM traps were set up throughout the night from 6 PM to 7:30 AM, with a single collection in the morning (Table 1).

**Table 1:** Trapping calendar: Number of nights and schedule.

	2017			2018		
	September 12-15 (2 nights and 1 full night)	October 09-12 (3 nights)	November 06-09 (3 full nights)	August 21-24 (3 nights)	September 10-14 (3 nights)	October 01-04 (3 nights)
Transect						
Health centre	Every night	Every night	Every night	Every night	Every night	Every night
Mango tree	Every night	Every night	Every night		1 night	1 night
Community carbet		Every night	Every night		Every night	Every night
Other MM traps						
Water tower					Every night	Every night
Slash-and-burn fields					1 night	2 nights
Landing stage			Every night	1 night	1 night	

Nights: from 6 PM to 7 AM in 2017 and from 5 PM to 8 AM in 2018, with hourly collections, except between midnight and 5 AM, with only one collection at 3 AM.

Full nights: from 6 PM to 7 AM with one collection at 7 AM.

Mosquito Magnet traps with octenol bait were used, based on their capacity to attract *Anopheles* species [27,28]. All mosquito specimens were morphologically identified to species level using regional identification keys [29]. Each specimen’s trap location, date, and time of collection were recorded. The study focused on the abundance dynamics of species of interest, specifically those known as disease vectors or human nuisances.

To analyse the data, the number of mosquitoes collected between midnight and 3 AM was divided by three, and the number collected between 3 AM and 5 AM was divided by two, to obtain the number of mosquitoes per hour from midnight to 5 AM.

Among the 60 traps night set up, only one trap was out of order for an entire night (slash-and-burn fields, October 2018). This same trap was out of order for two hours in the middle of the night in September 2018. The mango tree trap and the water tower trap were out of order for one hour one night in the early morning in September 2018. The landing stage trap was out of order for two hours in the early evening in August 2018 and one hour in the early evening in September 2018. For the traps that failed during one or two-hours time slots, the number of mosquitoes for that time slot was estimated by taking the mean of mosquitoes trapped in the one-hour time slot before and the one-hour time slot after the malfunction. Data from the trap that failed throughout the night were excluded from the analysis.

### Statistical analysis

The different variables were first described separately and then combined to describe potential at-risk situation (exposure without protection). Bivariate analyses were also performed to compare malaria carriers and non-carriers in 2017 and 2018 in terms of exposure and protection. Categorical variables were described variables as frequencies and percentages, while quantitative variables were described as medians and interquartile range (IQR).

Comparisons between exposures, protections and knowledge were conducted using Chi-2 test (or Fisher’s exact test) for categorical characteristics and Student’s t-test (or Mann-Whitney test) for quantitative measurements.

*Anopheles* spp. relative abundance indicates the proportion of *Anopheles* mosquitoes caught during a one-hour time slot out of the total *Anopheles* spp. caught throughout the entire night by the MM traps.

A generalized additive mixed model (GAMM) with logistic regression stratified by year was built to assess the effect of both exposure and protection on malaria carriage for each year. A random effect on house localization was added as a proxy of family to consider the effect of possible unassessed common behaviours in the same family. A Gaussian process using a power exponential covariance function was applied to the geographical coordinates of participants’ homes to take into account the potential effect of the proximity of homes in the spread of the disease [30]. A spline was used to interpolate the quantitative data in the model (age, potential ineffectiveness of bed nets and distance from the forest). All the variables assessed in the bivariate analysis were used in the model, as they are all suspected to be potentially linked to malaria carriage.

The statistical analyses were performed using R software version 4.1.2. (Copyright 2022, R Foundation for Statistical Computing). The maps in the figures were created using QGIS, version 2.18.28 (Open Source Geospatial Foundation Project, Beaverton, OR, USA).

## Results

### Entomological results

A total of 10,494 mosquitoes were caught across all MM traps. Among them, *Coquillettidia* spp. and *Anopheles* spp. were the most frequently captured species, 78% (n = 8,206) and 20% (n = 2,113), respectively. *Anopheles darlingi* accounted for 99.7% (n = 2,106) of the *Anopheles* mosquitoes caught (S1 Table).

*Anopheles darlingi* was detected in all traps along the transect, with a higher abundance near the forest in September and October 2018 (Table 2). The seasonal abundance pattern of *An. darlingi* varied between 2017 and 2018 at the health centre trap (the only constant trap throughout the study). In 2017, the highest abundance was observed in September followed by a decrease in October. Conversely, fewer *An. darlingi* were captured in September 2018, but there was an increase in October 2018 (Table 2). No *An. darlingi* were found in the slash-and-burn fields.

**Table 2:** Daily mean number and hourly evening median number (from 6PM to midnight) of *Anopheles darlingi* per month and per trap.

			September 2017		October 2017		November 2017	
			Monthly mean	Evening Hourly median (IQR)	Monthly mean	Evening Hourly median (IQR)	Monthly mean	Evening Hourly median (IQR)
**Transect**								
Health centre			77	9 (5.5-12.5)	18	1 (0-1.8)	0	/*
Mango tree			122	17.5 (5.8-21.5)	/	/	1	/*
Community carbet			/	/	7	1 (0-1)	1	/*
	**August 2018**		**September 2018**		**October 2018**			
	**Monthly mean**	**Evening Hourly median (IQR)**	**Monthly mean**	**Evening Hourly median (IQR)**	**Monthly mean**	**Evening Hourly median (IQR)**		
**Transect**								
Health centre	97	8 (4-12)	43	3 (1-6)	64	6 (2.5-8)		
Mango tree	/	/	17	3 (2.3-3)	30	2.5 (2-3.8)		
Community carbet	/	/	16	1 (0.8-2.3)	26	2 (0.3-3)		
**Other traps**								
Slash-and-burn fields	/	/	0	0 (0-0)	0	0 (0-0)		
Water tower	/	/	84	9 (3-13.5)	79	8 (6-13.5)		
Landing stage	4	0.5 (0.1-0.9)	17	2 (1-3.8)	/	/		

* No hourly data was available in November 2017.

**Fig 2:**
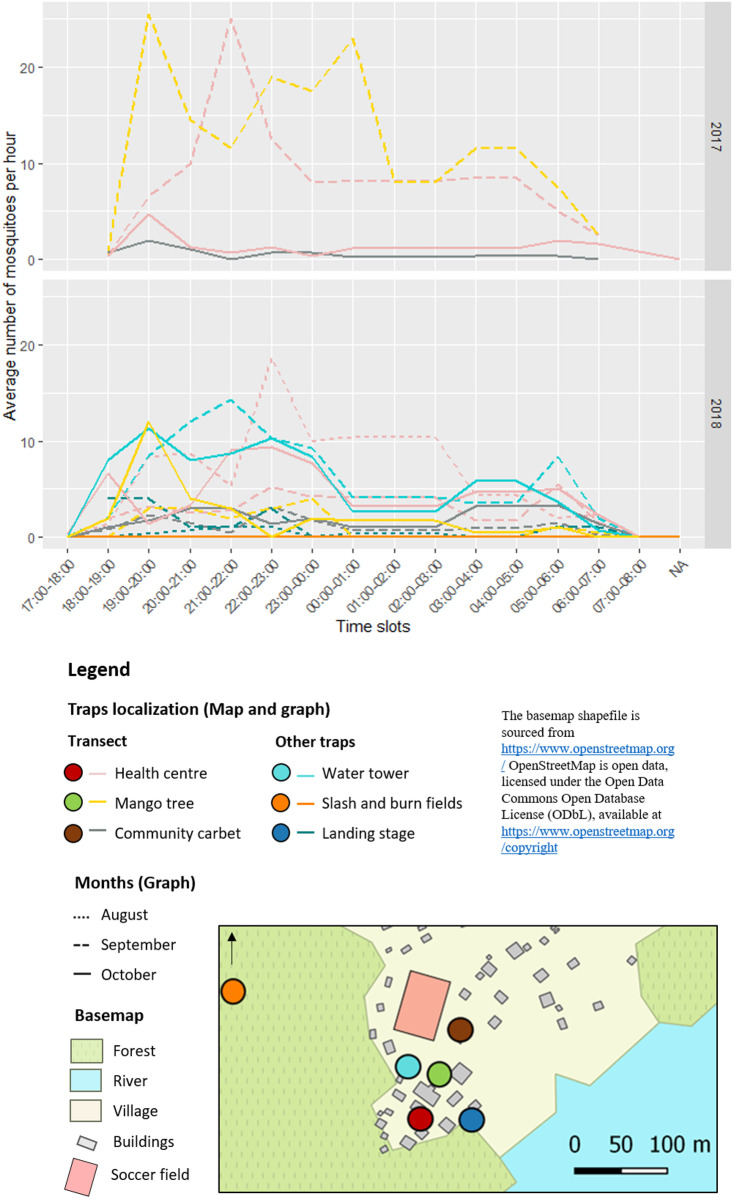
Average number of *Anopheles darlingi* per hour by Mosquito Magnet (MM) trap and month year in Trois-Palétuviers, French Guiana. Data for November 2017 are not available on an hourly basis. Reading explanation: No MM traps were used between 17:00 (5 PM) and 18:00 (6 PM) in 2017. In contrast, MM traps were used during the same time slot in 2018, but no *Anopheles darlingi* were caught in any of the traps, regardless of their location. The basemap shapefile is sourced from https://www.openstreetmap.org/ OpenStreetMap is open data, licensed under the Open Data Commons Open Database License (ODbL), available at https://www.openstreetmap.org/copyright.

*Anopheles darlingi* were caught throughout the night, with higher abundance in the evening hours (from 7 PM to midnight, depending on the location and the month) and a slight increase in abundance in the early morning (around 5–6 AM) (Fig 2). No *An. darlingi* were caught before 6 PM. The majority of *An. darlingi* caught between 6 PM and 7 PM were observed particularly in 2018 in traps located near the forest (Fig 2).

### Population characteristics

#### Sociodemographic data, pathway and access to care and malaria knowledge.

A total of 182 participants were included in the survey. Among them, 9 did not participate in the 2018 evaluation (6 had relocated to another area, 1 was absent and 2 could not be located). The median age of the study population was 12.5 years in 2017 (IQR [5;27]; range 0–71 years), and 58% (n = 106) were male. The median household density of the study population was 8 inhabitants (IQR [5;11]; range 2–17 inhabitants) (Table 3).

**Table 3:** Sociodemographic characteristics of the study population.

Characteristics	Total number of persons*
**Total**	182 (100%)
**Male**	106 (58%)
**Age group (years)**	
0 – 3	37 (20%)
4 – 11	50 (27%)
12 – 17	22 (12%)
18 – 25	22 (12%)
26 – 45	39 (21%)
> 45	12 (7%)
**Nationality**	
French	140 (77%)
Brazilian	42 (23%)
**Social security system**	
French or Brazilian Social Security System	143 (78%)
No Social Security	28 (15%)
No answer	11 (6%)
**Amerindian ethnic group**	154 (85%)
**Number of people in the household**	8 (5, 11)
**Number of years spent in Trois-Palétuviers**	8 (3, 17)

SGO: Saint-Georges.

*N (%), Median (IQR).

The adult participants’ occupations included outdoor activities (38%, n = 28) (i.e., slash-and-burn farming (23%, n = 17), sailing/fishing (8%, n = 6) or hunting (7%, n = 5)), homemakers (29%, n = 21) and informal work (26%, n = 19). Five participants were retired (7%, n = 5).

In terms of access to care, 15% (n = 28) of the participants did not have any social and health insurance (Table 3). Most participants went to the SGO heath centre for their last fever (84%, n = 152). The others mainly went to Oiapoque (8%, n = 15) and only 2% (n = 3) went to Taparabu, the nearest Brazilian health centre.

Regarding malaria knowledge among participants aged 7 and over (n = 127), 72% (n = 92) had already heard about malaria and 55% (n = 70) knew that malaria is transmitted by mosquitoes. Regarding knowledge of vector control methods, 43% (n = 55) of all participants agreed that bed nets can prevent malaria transmission, 15% (n = 19) mentioned skin repellents, 8% (n = 10) mentioned indoor insecticides, 6% (n = 7) mentioned medication and less than 5% mentioned plants (n = 6), long clothing (n = 6), emptying water bowls (n = 4) and spraying in the village (n = 2). Young adults were the most aware of malaria and vector control methods among the population (S2 Table).

#### Risk of exposure and protection description.

##### Exposure:

Participants were potentially exposed through different activities outside the village, such as slash-and-burn farming at night (17%, n = 31), hunting at night (12%, n = 22), fishing at night (8%, n = 15), visiting gold mining sites (7%, n = 12) or travelling in high-risk areas (41%, n = 74). They were also potentially exposed inside the village by waking up before 7 AM (62%, n = 107), playing football after 6 PM (13%, n = 23) or watching TV without bed nets (72%, n = 124) (Table 4). Hunting and fishing at night, visiting gold mining site, travelling in high risk areas and playing football were more frequent among teenagers and adults. In contrast, children were more likely to wake up before 7 AM (S3 Table).

**Table 4:** Potential exposure inside and outside the village and protection used among participants.

Characteristics	Total number of persons*
**Total**	182 (100%)
**Exposure outside the village**	
**Slash-and-burn farming at night**	31 (17%)
**Hunting at night**	22 (12%)
**Fishing at night**	15 (8%)
**Visiting gold mining site**	12 (7%)
**Travelling in high-risk area**	74 (41%)
**Exposure inside the village**	
**Waking up before 7 AM**	107 (62%) *NA = 9*
**Playing football after 6 PM**	23 (13%) *NA = 9*
**Watching TV without bed nets**	124 (72%) *NA = 9*
**Distance between home and nearest forest edge (meters)**	41 (11, 86)
**Individual protection**	
**Bed nets**	180 (99%)
**Factors of bed net ineffectiveness**	1 (1, 2)
**Window screens**	7 (4%)
**Skin repellent**	57 (32%)
**Indoor insecticides**	114 (63%)

*N (%), Median (IQR)

NA: not available (missing data); AM: Ante Meridiem; PM: Post Meridien.

##### Protection:

Almost all the participants used bed nets (99%, n = 180) while 63% (n = 114) used indoor insecticides sometimes or often, and 32% (n = 57) used skin repellents sometimes or often.

###### Protection effectiveness:

Regarding the origin of bed nets, 91% (n = 163) were obtained through free distribution. The most prevalent potential factor of bed nets ineffectiveness was sun drying (72%, n = 123), followed by inadequate washing frequency (38%, n = 65) and inadequate washing mode (18%, n = 32). Additionally, 15% (n = 26) of the participants reported having bed nets with holes, 6% (n = 10) were not impregnated with insecticides, and 6% (n = 11) were over 2 years old (S4 Table).

Looking at all of the potential factors of bed net ineffectiveness, 12% (n = 20) of participants bed nets were effective, while 44% (n = 75) had one potential ineffectiveness factor, 28% (n = 48) had two factors, 11% (n = 19) had three factors, 4% (n = 6) had four factors and 1% (n = 1) had five potential factors of ineffectiveness. Eleven participants had missing data for at least one factor.

###### Protection motivation:

The use of skin repellent was not significantly increased by the fact that participants already heard about malaria or knew that mosquitoes could transmit malaria. However, it was significantly increased by the belief that skin repellents could prevent malaria transmission (58% of the participants aged 7 and over who thought that skin repellent could prevent transmission used skin repellent vs 28% of those who did not think that it could prevent transmission, p = 0.010). No significant differences were found for the use of indoor insecticides (S5 Table).

The use of skin repellents was not significantly associated with most of the potential exposures assessed in the survey. However, lower use of skin repellent was reported by participants who went to slash-and-burn fields at night (16% used repellent) compared to those who did not (34% used repellent, p = 0.045). Conversely, higher use of skin repellent was reported among participants who had travelled to high-risk areas (43% used repellent) compared to those who had not (23% used repellent, p = 0.004) (S6 Table).

###### At-risk situations (exposure without protection):

**Fig 3:**
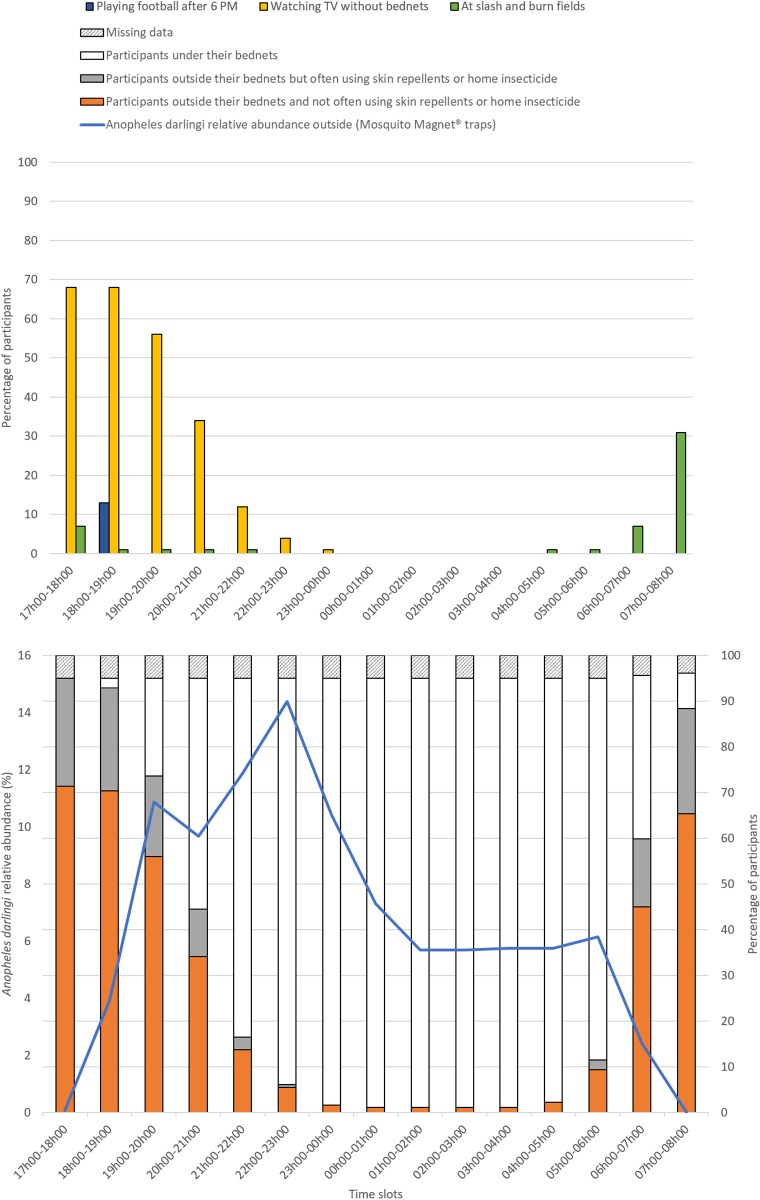
Human exposure to *Anopheles darlingi* and nighttime protective measures deployed in Trois-Palétuviers, French Guiana.

The highest exposure of participants to night-time bites was observed during periods of high relative abundance of *An. darlingi*. Specifically, 56% of the participants were not under their bed nets and not using skin repellent or indoor insecticides between 7 PM and 8 PM, while the relative abundance of *An. darlingi* was 11%. Most participants were sleeping under their bed nets from 10 PM to 6 AM. On average, participants were more exposed and less protected in the evening compared to the morning (Fig 3).

### Epidemic dynamics

**Fig 4:**
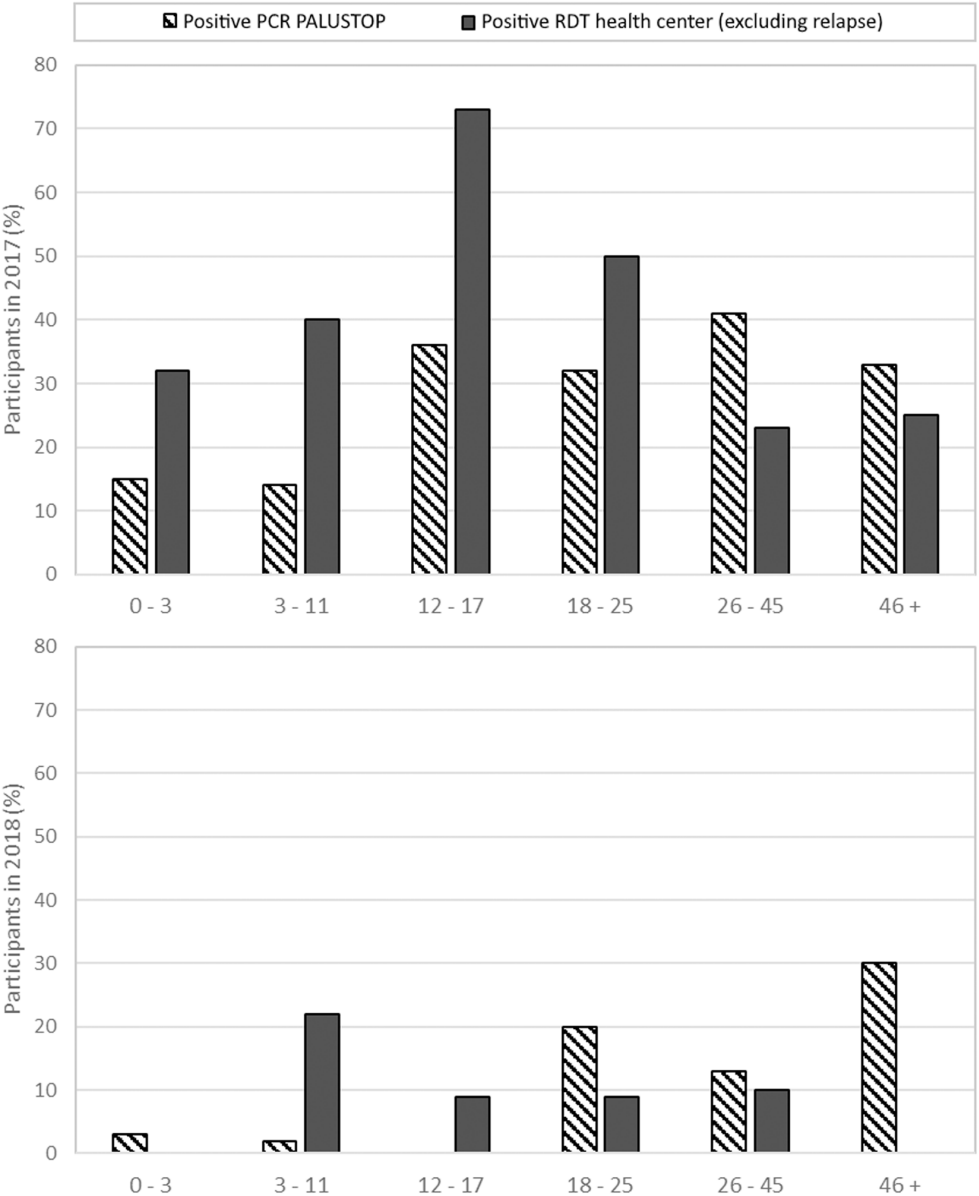
Distribution of *Plasmodium spp*. episodes (health centre) or positive PCR tests (PALUSTOP) in 2017 (top) and 2018 (bottom) by age group in Trois-Palétuviers, French Guiana.

In 2017, 39% (n = 71) of the participants were diagnosed with at least one *Plasmodium* spp. episode (excluding relapses) at the SGO health centre, whereas in 2018, they were 10% (n = 19). During the first PALUSTOP survey in 2017, 25% (n = 46) of the participants had a *P. vivax* positive PCR result and 1% (n = 1) had a *P. falciparum* positive PCR result (3 missing PCR results). In the second PALUSTOP survey in 2018, 7% (n = 13) of the participants had a *P. vivax* positive PCR result and 1% (n = 1) of the participants had a *P. falciparum* positive PCR result (10 missing PCR results). Children were more frequently diagnosed with at least one *Plasmodium* spp. episode at the SGO health centre, whereas they had less *Plasmodium* spp. positive PCR results during PALUSTOP surveys (Fig 4).

**Fig 5:**
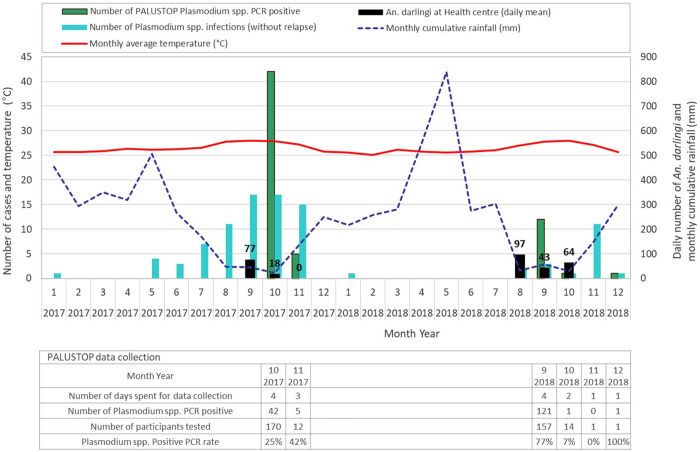
Dynamics of malaria cases, *Anopheles darlingi* caught at the health centre, average monthly temperature and cumulative monthly rainfall in 2017 and 2018, Trois-Palétuviers, French Guiana. Mean monthly temperature and cumulative monthly rainfall data were collected for the study period using Google Earth Engine using RGEE package from R software [31,32]. Rainfall data were obtained from the CHIRPS dataset, developed by the Climate Hazards Group at the University of California, Santa Barbara (UCSB) [33]. Temperature data come from the ERA5 dataset, which integrates multiple observations into a model that tracks climate change on an almost daily basis [34].

The dynamics of cases diagnosed at the SGO health centre showed similarities in 2017 and 2018, with the majority of the cases identified between August and November. However, the 2017 epidemic started earlier and the *An. darlingi* density decreased earlier that year. Both *Plasmodium* spp. epidemics coincided with periods of lower cumulative rainfall and higher average monthly temperatures (Fig 5).

### Exposure, protection and malaria carriage per year

In 2017, 51% (n = 92) of the participants experienced at least one episode of malaria diagnosed at SGO health centre (excluding relapses) or/and had a positive PCR test during the PALUSTOP first survey, while they were 16% (n = 30) in 2018.

The age of participants was the only factor associated with malaria in 2017 (p = 0.013), with a higher median age observed among malaria carriers compared to non-malaria carriers (16 versus 9 years old respectively). While in 2018 the factors were slash-and-burn farming at night (p < 0.001), playing football after 6 PM (p = 0.033) and residing closer to the forest (p = 0.028) (Table 5).

**Table 5:** Exposure and protection compared to malaria carriage per year.

Characteristics	No malaria in 2017 (%)*	Malaria in 2017 (%)*	p-value	No malaria in 2018 (%)*	Malaria in 2018 (%)*	p-value**
**Total**	90 (100%)	92 (100%)		152 (100%)	30 (100%)	
**Sociodemographic variables**						
**Age**	9 (3, 26)	16 (7, 29)	**0.01**	12 (4, 26)	18 (9, 28)	0.08
**Female**	34 (38%)	42 (46%)	0.30	67 (44%)	9 (30%)	0.20
**French nationality**	67 (74%)	73 (79%)	0.40	116 (76%)	24 (80%)	0.70
**Exposures outside the village**						
**Slash-and-burn farming at night**	12 (13%)	19 (21%)	0.20	19 (12%)	12 (40%)	**< 0.001**
**Hunting at night**	9 (10%)	13 (14%)	0.40	16 (11%)	6 (20%)	0.20
**Fishing at night**	7 (8%)	8 (9%)	0.80	10 (7%)	5 (17%)	0.08
**Visiting gold mining sites**	4 (4%)	8 (9%)	0.20	9 (6%)	3 (10%)	0.40
**Travelling in high-risk area**	36 (40%)	38 (41%)	0.90	60 (39%)	14 (47%)	0.50
**Exposures inside the village**						
**Waking up before 7 AM**	53 (62%) *NA = 5*	54 (61%) *NA = 4*	0.90	84 (59%) *NA = 9*	23 (77%) *NA = 0*	0.07
**Playing football after 6 PM**	8 (9%) *NA = 5*	15 (17%) *NA = 4*	0.14	15 (10%) *NA = 9*	8 (27%) *NA = 0*	**0.03**
**Watching TV without bed nets**	62 (73%) *NA = 5*	62 (70%) *NA = 4*	0.70	101 (71%) *NA = 9*	23 (77%) *NA = 0*	0.50
**Exposure at home**						
**Distance from forest (meters)**	46 (14,104)	41 (4, 86)	0.10	46 (11, 94)	32 (4, 46)	**0.03**
**Protection**						
**Skin repellent sometimes or often**	33 (37%)	24 (26%)	0.12	48 (32%)	9 (30%)	0.90
**Factors of bed net ineffectiveness**						
**0**	8 (9%)	12 (14%)	0.40	14 (10%)	6 (21%)	0.20
**1**	38 (44%)	37 (44%)		66 (46%)	9 (32%)	
**2**	24 (28%)	24 (28%)		37 (26%)	11 (39%)	
**3**	13 (15%)	6 (7%)		18 (13%)	1 (4%)	
**4+**	3 (4%) *NA = 4*	6 (7%) *NA = 7*		8 (6%) *NA = 9*	1 (4%) *NA = 2*	

*N (%), Median (IQR).

**Pearson’s Chi-squared test, Kruskal-Wallis rank sum test, Fisher’s exact test.

NA: not available (missing data). Factors of bed net ineffectiveness: score based on the sum of potential factors of ineffectiveness, defined as follow: Bed nets not impregnated with insecticides, bed nets with holes, bed nets over 2 years old, inadequate washing (using washing machine or not washing them),inadequate washing frequency (more or less than every month) and sun drying.

**Fig 6:**
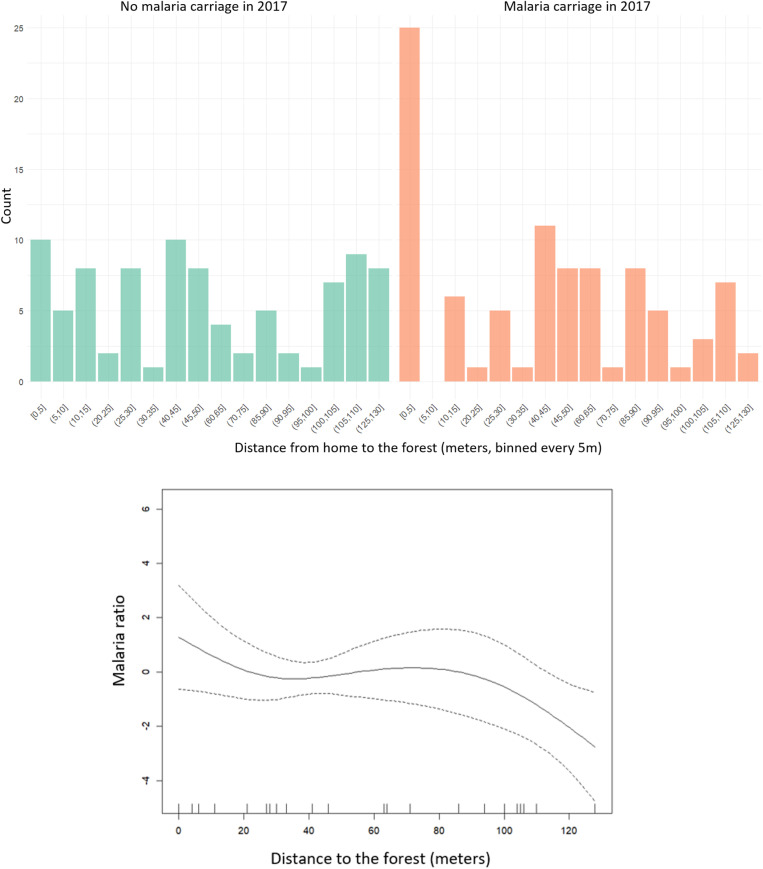
Barplot of the distance to the forest according to malaria status in 2017 with its Generalized additive model result (p = 0.023).

Considering all the variables in the multivariate analysis, the only factor associated with malaria in 2017 was the proximity to the forest (Fig 6), with a lower risk for participants living far from the forest (p = 0.023). In 2018, only slash-and-burn farming at night (OR=3.70 IC95 [1.11-12.29]) and playing football after 6 PM (OR=4.18 IC95 [1.11-15.72]) were associated with malaria.

## Discussion

### Key results

The focus on a specific site enables a detailed description of local interactions between human populations and malaria vectors, as well as the impact of environmental factors. The integration of these human, vector, and environmental factors offered a holistic, multidisciplinary perspective, essential to understand the complexity of at-risk situations for malaria transmission.

The primary vector identified in our study was *An. darlingi*, as expected in French Guiana [16,35–40]. Surprisingly, traps located near the forest captured more *An. darlingi,* while traps situated in slash-and-burn fields did not capture any *An. darlingi.*

In our study, vector control measures appeared inadequate, as *An. darlingi* were found active before bedtime, suggesting that bed nets alone may not be sufficient. Additionally, bed nets may not be as effective as anticipated, mainly due to sun drying habits. Skin repellents and indoor insecticides are not always used, particularly those that could offer protection outside the home, where inhabitants may be exposed while engaging in activities such as playing football, fishing, hunting or staying in slash-and-burn fields.

Overall, our findings suggest interconnections between the environment, mosquitoes dynamic and the dynamics of malaria cases, with concurrent variations observed across these phenomena during the 2017 and 2018 epidemics.

### Interpretation

**Fig 7:**
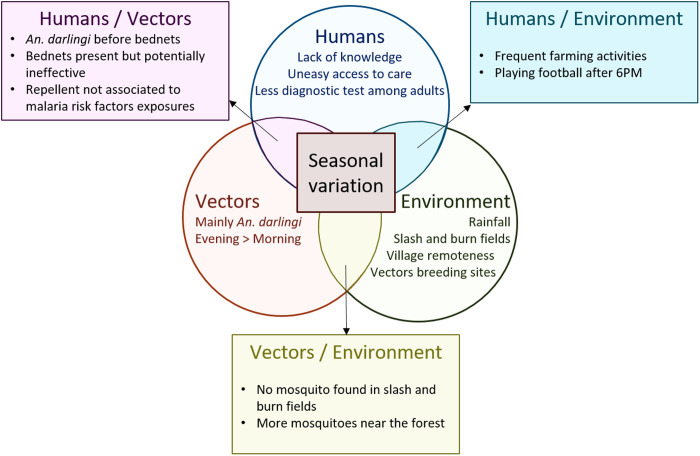
Study findings regarding potential risk factors for malaria: One Health summary.

Despite the small size of the area investigated, our study found a geographical disparity in *An. darlingi* abundance. We did not capture any *An. darlingi* in slash-and-burn fields, contrary to expectations given the correlation between slash-and-burn farming and malaria carriage observed in our study and other studies in the region [22]. Possible explanations include non-measured confounding factors, exposure along the path to fields, unmeasured vector presence during daytime, the presence of other *Anopheles* spp. not attracted to MM traps, or their potential lack of sensitivity for *An. darlingi* (meaning the vector is present but in lower abundance) [37,40,41] (Fig 7). *Anopheles* species such as *An. nuneztovari s.l.* or *An. oswaldoi s.l.* have been identified as potential malaria vectors and have been found in forested areas in French Guiana [40–43]. Moreover, slash-and-burn are seasonal activities that can change the landscape of the fields from one month to another [44]. Finally, the other studies in the area that found *Anopheles* species in fields, found higher abundance in the neighbouring villages [45].

The unexpected higher abundance of *An. darlingi* near the forest may be influenced by factors such as population density, oviposition site availability, or proximity to food sources. We did not find any similar result in the literature; however, higher distance to the forest and the absence of vegetation within 50 meters around habitations have already been found to be associated with a lower risk of malaria infection in an upstream village [43]. Moreover forest fragmentation has been shown to increase malaria and the occurrence of *Plasmodium*-infected mosquitoes in peridomestic habitats in Brazil [46], while deforestation has been linked to increased malaria risk in the Amazon, with different hypotheses on the role of *An. darlingi* or other vectors in that context [47].

The mosquito population community was very different between 2017 and 2018, with a proliferation of *Coquillettidia albicosta* in 2018, and a more rapid decrease of *Coquillettidia* spp. in 2018 compared to *An. darlingi* (S7 Table). High abundances of mosquitoes from the genus *Coquillettidia* are a serious nuisance to humans because of their anthropophilic behaviour. During the data collection, we observed that the inhabitants used protective measures (smoke production, sheltering in homes, etc.) against *Coquillettidia* spp. nuisance when attacks began at dusk. *Anopheles darlingi* bites, which began before *Coquillettidia albicosta* bites (field observation, not visible in results of hourly collected MM traps), did not trigger this protective behavior as visibly [48]. The use of protective measures should be further investigated, particularly focusing on the impact of nuisance and/or fear of disease on the motivation to use them.

In our multivariate analysis, only a few exposures were found to be linked to malaria each year (i.e., visiting slash-and-burn fields at night and playing football after 6PM). Notably, trips to high-risk areas, which have been described as linked to these epidemics, were not associated with malaria in our study. This could be explained by the large number of people contaminated and the annual scale of the prevalence chosen for this study, which may hide the role of trips to high-risk areas as a trigger for the epidemics. Indeed, these trips might be more responsible for the resurgence of the epidemic and not necessarily contribute as much to the spread of the disease compared to local contamination, especially during the evening when not enough people were using protective measures [14,22].

Bed nets are the primary tool used worldwide to protect from nocturnal mosquito bites and fight malaria [49,50]. Education on bed net use, particularly the washing process, needs to be improved, and discussions about its feasibility with the inhabitants are essential [51–53] (Fig 7).

Regarding access and pathways to care, we found that the age distribution of malaria cases in the neighbourhood showed a higher rate of malaria infections diagnosed at the SGO health centre among the youngest participants, while the test-and-treat campaign found a higher rate of malaria carriers among older participants. These observations are consistent with a greater demand for care among the young, whether due to a higher rate of symptoms (due to low immunity), perceived malaria risk in children, or easier access to care for middle school pupils who commute daily to the SGO city centre for school. Moreover, the delay in consultation was also higher in the PALUSTOP study in that village due to its remoteness.

Regarding the temporal disparity in *An. darlingi* abundance throughout the months, previous studies have described a link between environmental factors and *An. darlingi* oviposition, with different observations and underlying mechanisms depending on the area. Nevertheless, the increased abundance of *Anopheles* spp. during the declining phase of the rainy season was concordant with previous observations in the area, likely due to the reduction in river flow and higher levels of stagnant water [13,38,54,55]. Moreover, despite *An. darlingi* generally being more abundant in the evening compared to the early morning in our study, we observed monthly variations in abundance throughout the night for all traps. Some traps showed an early peak one month and a later peak or two peaks the following month. These results are consistent with the well-known plasticity and highly adaptive behaviour of *An. darlingi* to its environment, making its control more challenging and requiring constant surveillance to adjust public health messages and strategies [56].

### Public health implications

The lifestyle of inhabitants, combined with local *Anopheles* spp. dynamics and environmental factors, increases their risk of malaria exposure. Therefore, protection and education are crucial to prevent malaria transmission. Our study identified potential shortcomings in effectiveness within the neighbourhood, which could be addressed through targeted measures.

The potential decrease in the effectiveness of bed nets could be mitigated by maintaining free distribution every two years to ensure the efficacy of protection strategies at night. Additionally, education campaigns on how to use and maintain them are essential (e. g., avoiding sun drying, proper washing procedures, etc). However, protection strategies should be enlarged to prevent mosquito bites in the evening and early morning when individuals are not under bed nets. Various tools have been developed for this purpose, including window screens, terrace nets, and anti-mosquito diffusers for indoor or around-the-house use, which are highly adapted to the lifestyle in Trois-Palétuviers. Skin repellents, anti-mosquito socks and hammocks with nets are also available for outdoor activities such as fishing, hunting, playing football or sleeping in slash-and-burn fields, but further research is still needed to assess their effectiveness and safety, especially for long-term use in younger children and pregnant women [57–59]. Moreover, those tools are not well adapted and are expensive for this specific population, considering the low rate of use among our participants. New tools are needed to enhance protection outside the home. These tools must be adapted to the evolving housing structures in the area, particularly with the development of brick closed houses. Distribution and training on the proper use of these tools are essential to ensure their effectiveness. Furthermore, specific interventions such as seasonal screening and treatment strategies could be used in the meantime to fight malaria in areas with recurrent epidemics and insufficient protective tools.

The implementation of new preventive strategies should be tailored to the behaviours and needs of local communities, especially by promoting additional protective methods. These strategies must take into account the cultural and economic constraints specific to each community, by facilitating co-construction with communities and involvement of peers in the process. Educating people about mosquito behaviour, distinguishing *Anopheles* from other mosquitoes, and how to protect themselves, and conveying a clear message that the absence of perceived nuisance does not imply the absence of risk, is also crucial. This ensures that protective measures are not only used to prevent annoyance but also during periods when mosquito nuisance decreases but *Anopheles* spp. mosquitoes remain active, such as in October and November.

In addition to protective measures, improved access to healthcare must be implemented to promptly reach children but also adults. Education on the importance of being tested, even if symptoms are mild, is essential for community protection. Test-and-treat strategies should be reinforced to detect asymptomatic cases. In remote areas like this one, community health workers play a vital role in reaching all inhabitants and explaining the importance of preventive measures. They could also contribute to increasing testing rates by delivering test at home.

The dynamic variation observed between 2017 and 2018, consistent with the literature, underscores the importance of reactive surveillance for entomological, environmental and malaria case dynamics to tailor personalized interventions as effectively as possible, as well as targeting the relevant season for prevention campaigns and community health worker actions [60,61]. This surveillance could also allow malaria actors to understand the environmental and local context better and how it influences human and vector behaviours, thereby affecting malaria transmission, in order to eliminate it more efficiently. Systematic entomological surveillance is particularly needed in areas with recurrent high transmission levels.

Finally, our findings highlight the importance of an interdisciplinary approach to analyse the interactions between each compartment of malaria transmission. These approaches are essential in malaria research but are difficult to implement in real life, making concurrent human, entomological, and environmental data rare. In our study, the active malaria actor network, as well as the village size and location (isolated but accessible), allowed us to deeply investigate these three compartments at the same time. However, this project was funded by different grants for each compartment rather than as a single whole, project.

### Limitations

Our study has several limitations.

First, the diversity of entomological data was limited by logistical and cost constraints – transport of the traps by pick-up and then by pirogue to the village –, which restricted the number of traps deployed. The safety conditions during night trapping (risk of bites or injuries) made it necessary to concentrate the transect in a portion of the village and required a second team to manage only the trap deployed in the slash-and-burn fields. Moreover, we opted for a transect methodology coupled with exploration of other locations for only one month to optimize data collection within material constraints. While this approach provided extensive information on the transect throughout the two epidemic periods sampled, it did not allow us to utilize all the data collected in other locations. Additionally, the lack of data at the beginning of 2017 and 2018 epidemics and at the end of the 2018 epidemic obscured part of the mosquito dynamics during these periods. The heterogeneity of data collection, with some traps operating for only one night per month while others operated for three to four nights throughout the study period, may also have reduced the relevance of comparisons between traps and times.

Second, in terms of malaria case collection, the decision to collect cases at the SGO health centre may have underestimated the true number of malaria cases diagnosed in the neighbourhood, as some cases may have been diagnosed in Brazil. However, our analysis of access to care showed that 84% of participants visited the SGO health centre for their last fever, which provides some reassurance regarding the completeness of our data. Moreover, the use of both health centre and PALUSTOP data (using PCR, a more sensitive diagnostic tool), allows a global overview of the malaria situation in the area, covering both symptomatic and asymptomatic cases, especially with the very high PALUSTOP intervention coverage. Finally, the exclusion of relapses may introduce bias, as some diagnosed cases may actually be relapses of asymptomatic infections, and some cases categorized as relapses may actually be new contaminations. Nevertheless, we deemed this strategy to be the best option for assessing at-risk exposure situations.

Third, extrapolating cross-sectional behavioural data should be approached with caution, particularly with regard to exposure and protective measures, as behaviour can vary over time. However, these data were collected during the malaria season, which likely increases the reliability of the collected data as behaviour during this time may be more consistent.

Last, the choice to include all exposure variables as well as socio-demographic data in the model may have induced collinearity and potentially biased the final results. This could explain the change in results between the bivariate and model analyses. Nevertheless, we attempted to analyse the data by year and age (as age is the variable most strongly associated with other variables) and found similar results: in 2017, no significant factors were identified for adults, while distance from the forest and location were relevant for children; in 2018, no significant factors were found for adults, and only football was significant for children, although slash-and-burn farming had a p-value of 0.067. Moreover, these preliminary analyses are more exploratory in nature, focusing on the description and comparison of the data rather than identifying associations. Given the small dataset and the absence of uncollected potential factors, the model’s robustness is limited.

## Conclusion

In conclusion, our study confirms the key role of *An. darlingi* as the primary malaria vector in this area of French Guiana and underscores the practical application of an interdisciplinary approach by integrating human, vectorial, and environmental insights. The observed higher activity of *An. darlingi* in the evening and the lack of effective protection during this period highlight the necessity for tailored malaria control strategies that reflect the local ecological context and human behaviours. The absence of *An. darlingi* in expected locations, such as slash-and-burn fields, points to the complex ecology of malaria transmission and underscores the need for a nuanced understanding of environmental influences. By adopting an interdisciplinary perspective, our research offers a comprehensive view that can inform targeted and effective public health interventions, thereby enhancing the prospects for malaria control and contributing to sustainable health outcomes.

## Supporting information

S1 Fig Data collection timeline. (TIF)

S1 Table Description of the mosquitoes caught in the Mosquito Magnet traps. (DOCX)

S2 Table Malaria knowledge by age group of participants. (DOCX)

S3 Table Potential exposure inside and outside the village among participants by age range. (DOCX)

S4 Table Potential factors of participants bed net ineffectiveness. (DOCX)

S5 Table Use of skin repellents or home insecticide among participants 7 years and older depending their knowledge. (DOCX)

S6 Table Skin repellent use depending exposition. (DOCX)

S7 Table Monthly mean number of *Anopheles darlingi* and *Coquillettidia* caught per day. (DOCX)

S1 File PALUSTOP 2017 questionnaire. (PDF)

S2 File PALUSTOP 2018 questionnaire. (PDF)

S3 File Entomological data. (CSV)

S4 File Investigations pictures. (PDF)
