# Supplementary figures and images for: Breaking down malaria outbreak: A multidisciplinary approach in a border village of French Guiana
Source: PLoS Negl Trop Dis. 2025 Jun 17;19(6):e0013096. doi: 10.1371/journal.pntd.0013096 (PMC12212878; doi:10.1371/journal.pntd.0013096)

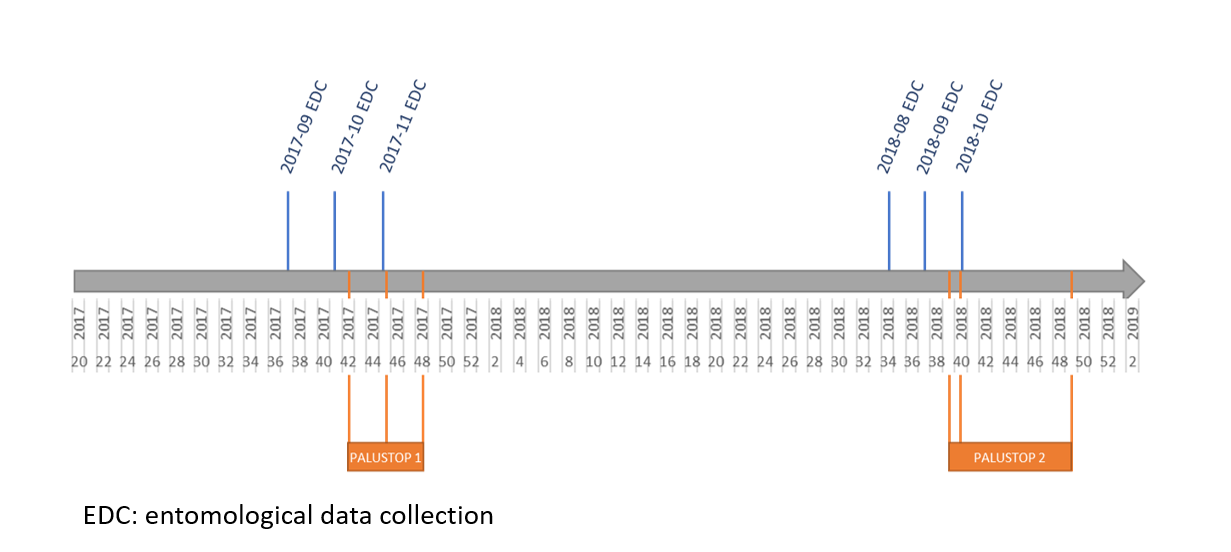

Supplement: S1 Fig — (TIF) [file pntd.0013096.s001.tif]
