# Supplementary material for: Breaking down malaria outbreak: A multidisciplinary approach in a border village of French Guiana
Source: PLoS Negl Trop Dis. 2025 Jun 17;19(6):e0013096. doi: 10.1371/journal.pntd.0013096 (PMC12212878; doi:10.1371/journal.pntd.0013096)
Supplement: S1 Table — (DOCX) [file pntd.0013096.s002.docx]

**S2 Table. Description of the mosquitoes caught in the Mosquito Magnet traps**

|  | **Number of mosquitoes** |
| --- | --- |
| **Total** | **10,494** |
| ***Coquillettidia* spp.** | 8,206 |
| ***Anopheles* spp.** | 2,113 |
| *Anopheles darlingi* | 2,106 |
| *Anopheles nuneztovari* | 1 |
| *Anopheles oswaldoi* | 1 |
| *Anopheles sp.* | 5 |
| ***Culex* spp.** | 122 |
| ***Mansonia* spp.** | 39 |
| ***Uranotaenia* spp.** | 9 |
| ***Wyeomyia* spp.** | 2 |
| ***Limatus*** **spp.** | 3 |
