# Supplementary material for: Breaking down malaria outbreak: A multidisciplinary approach in a border village of French Guiana
Source: PLoS Negl Trop Dis. 2025 Jun 17;19(6):e0013096. doi: 10.1371/journal.pntd.0013096 (PMC12212878; doi:10.1371/journal.pntd.0013096)
Supplement: S2 Table — (DOCX) [file pntd.0013096.s003.docx]

**S3 Table. Malaria knowledge by age group of participants**

|  | **Children**  **(7-11 years)**  Number (%) | **Teenagers**  **(12-17 years)**  Number (%) | **Young adults**  **(18-25 years)**  Number (%) | **Adults**  **(26-45 years)**  Number (%) | **Older adults**  **(>45 years)**  Number (%) |
| --- | --- | --- | --- | --- | --- |
| **Total** | 32 (100%) | 22 (100%) | 22 (100%) | 39 (100%) | 12 (100%) |
| **Heard about malaria** | 13 (41%) | 18 (82%) | 21 (95%) | 32 (82%) | 8 (67%) |
| **Think malaria is transmitted by mosquitoes** | 4 (12%) | 17 (77%) | 19 (86%) | 23 (59%) | 7 (58%) |
| **Think following items can prevent malaria** |  |  |  |  |  |
| Bed nets | 7 (22%) | 12 (55%) | 11 (50%) | 22 (56%) | 3 (25%) |
| Skin repellents | 1 (3%) | 4 (18%) | 7 (32%) | 7 (18%) | 0 (0%) |
| Home insecticides | 2 (6%) | 0 (0%) | 3 (14%) | 5 (13%) | 0 (0%) |
| Medication | 1 (3%) | 2 (9%) | 0 (0%) | 4 (10%) | 0 (0%) |
| Plants | 0 (0%) | 0 (0%) | 1 (5%) | 4 (10%) | 1 (8%) |
| Long clothes | 0 (0%) | 1 (5%) | 2 (9%) | 3 (8%) | 0 (0%) |
| Empty water bowls | 0 (0%) | 0 (0%) | 2 (9%) | 2 (5%) | 0 (0%) |
| Spraying in the village | 0 (0%) | 0 (0%) | 1 (5%) | 1 (3%) | 0 (0%) |
