# Supplementary material for: Breaking down malaria outbreak: A multidisciplinary approach in a border village of French Guiana
Source: PLoS Negl Trop Dis. 2025 Jun 17;19(6):e0013096. doi: 10.1371/journal.pntd.0013096 (PMC12212878; doi:10.1371/journal.pntd.0013096)
Supplement: S3 Table — (DOCX) [file pntd.0013096.s004.docx]

**S4 Table. Potential exposure inside and outside the village among participants by age range**

| **Age (years old)** | **0-6** | **7-11** | **12-17** | **18-25** | **26-45** | **46+** | **p-value** |
| --- | --- | --- | --- | --- | --- | --- | --- |
| **Total** | 55 (100%) | 32 (100%) | 22 (100%) | 22 (100%) | 39 (100%) | 12 (100%) |  |
| **Slash-and-burn farming at night** | 7 (13%) | 7 (22%) | 4 (18%) | 1 (5%) | 8 (21%) | 4 (33%) | 0.2 |
| **Hunting at night** | 0 (0%) | 0 (0%) | 3 (14%) | 2 (9%) | 13 (33%) | 4 (33%) | **<0.001** |
| **Fishing at night** | 0 (0%) | 0 (0%) | 2 (9%) | 0 (0%) | 11 (28%) | 2 (17%) | **<0.001** |
| **Visiting gold mining site** | 0 (0%) | 0 (0%) | 2 (9%) | 2 (9%) | 7 (18%) | 1 (8%) | **0.003** |
| **Travelling in high-risk area** | 14 (25%) | 12 (38%) | 12 (55%) | 10 (45%) | 23 (59%) | 3 (25%) | **0.015** |
| **Waking up before 7 AM** | 25 (48%)  *NA=3* | 25 (78%) | 9 (45%)  *NA=2* | 10 (50%)  *NA=2* | 32 (82%) | 6 (60%)  *NA=2* | **0.002** |
| **Playing football after 6 PM** | 1 (2%)  *NA=3* | 6 (19%) | 4 (20%)  *NA=2* | 3 (15%)  *NA=2* | 7 (18%) | 2 (20%)  *NA=2* | **0.033** |
| **Watching TV without bed nets** | 35 (67%)  *NA=3* | 26 (81%) | 16 (80%)  *NA=2* | 11 (55%)  *NA=2* | 30 (77%) | 6 (60%)  *NA=2* | 0.3 |
| **Distance from the forest** | 46 (28, 94) | 46 (11, 86) | 36 (11, 86) | 52 (33, 106) | 46 (8, 75) | 4 (0, 26) | 0.065 |

N (%), Median (IQR)

NA: not available (missing data)
