# Supplementary material for: Breaking down malaria outbreak: A multidisciplinary approach in a border village of French Guiana
Source: PLoS Negl Trop Dis. 2025 Jun 17;19(6):e0013096. doi: 10.1371/journal.pntd.0013096 (PMC12212878; doi:10.1371/journal.pntd.0013096)
Supplement: S4 Table — (DOCX) [file pntd.0013096.s005.docx]

**S5 Table. Potential factors of participants bed nets ineffectiveness.**

| **Potential factors of ineffectiveness** | **Bed nets*** |
| --- | --- |
| **Total** | 180 (100%) |
| **Bed net not impregnated** | 10 (6%)  *NA=1* |
| **Bed net with holes** | 26 (15%)  *NA=1* |
| **Over 2 years old** | 11 (6%)  *NA=1* |
| **Inadequate washing (washing machine or no washing)** | 32 (18%) |
| **Inadequate washing frequency (more or less than every month)** | 65 (38%)  *NA = 8* |
| **Sun drying** | 123 (72%)  *NA = 8* |

*N (%)

NA: not available (missing data)
