# Supplementary material for: Breaking down malaria outbreak: A multidisciplinary approach in a border village of French Guiana
Source: PLoS Negl Trop Dis. 2025 Jun 17;19(6):e0013096. doi: 10.1371/journal.pntd.0013096 (PMC12212878; doi:10.1371/journal.pntd.0013096)
Supplement: S5 Table — (DOCX) [file pntd.0013096.s006.docx]

**S6 Table. Use of skin repellents or indoor insecticide among participants 7 years and older depending their knowledge**

|  | **Heard about malaria**  Number (%) | **Never heard about malaria**  Number (%) | **p-value*** |
| --- | --- | --- | --- |
| **Total** | 92 (100%) | 35 (100%) |  |
| Use skin repellents | 30 (33%) | 11 (31%) | 0.9 |
| Use indoor insecticide | 55 (60%) | 26 (74%) | 0.13 |

|  | **Think malaria is transmitted by mosquitoes**  Number (%) | **Doesn’t think malaria is transmitted by mosquitoes**  Number (%) | **p-value*** |
| --- | --- | --- | --- |
| **Total** | 70 (100%) | 57 (100%) |  |
| Use skin repellents | 26 (37%) | 15 (26%) | 0.2 |
| Use indoor insecticide | 41 (59%) | 40 (70%) | 0.2 |

|  | **Think skin repellents can prevent malaria** Number (%) | | **Doesn’t think skin repellents can prevent malaria** Number (%) | **p-value*** |
| --- | --- | --- | --- | --- |
| **Total** | 19 (100%) | 108 (100%) | |  |
| Use skin repellents | 11 (58%) | 30 (28%) | | **0.01** |

|  | **Think home insecticide can prevent malaria** Number (%) | | **Doesn’t think indoor insecticide can prevent malaria** Number (%) | **p-value*** |
| --- | --- | --- | --- | --- |
| **Total** | 10 (100%) | 117 (100%) | |  |
| Use indoor insecticide | 9 (90%) | 72 (62%) | | 0.093 |

*Pearson’s Chi-squared test, Kruskal-Wallis rank sum test, Fisher’s exact test
