# Supplementary material for: Breaking down malaria outbreak: A multidisciplinary approach in a border village of French Guiana
Source: PLoS Negl Trop Dis. 2025 Jun 17;19(6):e0013096. doi: 10.1371/journal.pntd.0013096 (PMC12212878; doi:10.1371/journal.pntd.0013096)
Supplement: S6 Table — (DOCX) [file pntd.0013096.s007.docx]

**S7 Table. Skin repellent use depending exposure**

|  | **Number of participants** | **Skin repellent users n(%)** | **p-value*** |
| --- | --- | --- | --- |
| **Exposures outside the village** |  |  |  |
| **Slash-and-burn farming at night** |  |  | **0.045** |
| Yes | 31 | 5 (16%) |  |
| No | 151 | 52 (34%) |  |
| **Hunting at night** |  |  | 0.6 |
| Yes | 22 | 8 (36%) |  |
| No | 160 | 49 (31%) |  |
| **Fishing at night** |  |  | 0.079 |
| Yes | 15 | 8 (53%) |  |
| No | 167 | 49 (29%) |  |
| **Visiting gold mining site** |  |  | 0.2 |
| Yes | 12 | 6 (50%) |  |
| No | 170 | 51 (30%) |  |
| **Travelling in high-risk area** |  |  | **0.004** |
| Yes | 74 | 32 (43%) |  |
| No | 108 | 25 (23%) |  |
| **Exposures inside the village** |  |  |  |
| **Waking up before 7 AM** |  |  | 0.9 |
| Yes | 107 | 33 (31%) |  |
| No | 66 | 21 (32%) |  |
| **Playing football after 6 PM** |  |  | 0.3 |
| Yes | 23 | 5 (22%) |  |
| No | 150 | 49 (33%) |  |
| **Watching TV without bed nets** |  |  | 0.8 |
| Yes | 124 | 38 (31%) |  |
| No | 49 | 16 (33%) |  |
| **Distance from the forest (meters)** |  |  |  |
| 0 | 30 | 6 (20%) | 0.10 |
| 0-10 | 10 | 4 (40%) |  |
| 10-30 | 30 | 7 (23%) |  |
| 30-70 | 51 | 23 (45%) |  |
| >70 | 61 | 17(28%) |  |

*Pearson’s Chi-squared test, Kruskal-Wallis rank sum test
