# Supplementary material for: Breaking down malaria outbreak: A multidisciplinary approach in a border village of French Guiana
Source: PLoS Negl Trop Dis. 2025 Jun 17;19(6):e0013096. doi: 10.1371/journal.pntd.0013096 (PMC12212878; doi:10.1371/journal.pntd.0013096)
Supplement: S7 Table — (DOCX) [file pntd.0013096.s008.docx]

**S8 Table. Monthly mean number of *Anopheles darlingi* and *Coquillettidia* caught per day**

|  |  | **September 2017** | **October 2017** | **November 2017** |
| --- | --- | --- | --- | --- |
| **Number of traps** | / | 2 | 2 | 3 |
| ***Anopheles darlingi*** | / | 198 | 25 | 2 |
| ***Coquillettidia albicosta* or *venezuelensis*** | / | 259 | 45 | 3 |
|  | **August**  **2018** | **September 2018** | **October 2018** |  |
| **Number of traps** | 2 | 6 | 5 | / |
| ***Anopheles darlingi*** | 98 | 151 | 179 | / |
| ***Coquillettidia albicosta* or *venezuelensis*** | 810 | 1142 | 106 | / |
