# Supplementary material for: Breaking down malaria outbreak: A multidisciplinary approach in a border village of French Guiana
Source: PLoS Negl Trop Dis. 2025 Jun 17;19(6):e0013096. doi: 10.1371/journal.pntd.0013096 (PMC12212878; doi:10.1371/journal.pntd.0013096)
Supplement: S1 File — (PDF) [file pntd.0013096.s009.pdf]

Questionnaire PALUSTOP CAP (remplis par les médiateurs DAAC – l'IDE et le médecin)

Q1: zone de texte
Nom du médiateur :

Q000: affichage date
Date ?

Q000bis: 1 choix
Nom du quartier :

Esperance 1
Esperance 2
Crique Onozo
Savane
3 Palétuviers
Blondin
Bambou
Autre

Q0bis: affichage décimal
Coordonnées GPS (UTM) (ex 544225) :

Q1b: 1 choix
Consentement signé par la personne interrogée ou son représentant légal :
Oui

Q2: 1 choix (si non passer à Q9)
Avez-vous déjà entendu parler de paludisme (Malaria) ?
Oui
Non

Q3: choix multiple
Si oui, par qui ?
Ecole
Médecin
Médecin traditionnel
Famille
Affiches
Amis
Journaux
Facebook
Radio
Télévision
Whattsapp
Autre

Q4lab: lecture seule uniquement
Pensez-vous avoir eu assez d'informations sur

Q4: 1 choix
la prévention du paludisme ?
Oui
Non

Q5: 1 choix
quoi faire quand on a le paludisme (Malaria) ?
Oui
Non

Q6: 1 choix
ce qu'est le paludisme (Malaria) ?
Oui
Non

Q6bis: 1 choix
Peut-on mourir du paludisme ?
Oui
Non
Ne sais pas

Q7: choix multiple
Quels sont les symptômes/signes physiques du paludisme ?
Mal à la tête
Frissons
Diarrhées
Douleurs abdominales
Fatigue
Courbatures
Fièvre
Jaunisse
Autre
Ne sais pas

Q8: choix multiple
A votre avis, qu'est-ce qui transmet le paludisme ?
L'air
Toucher une autre personne qui a le paludisme
L'eau avalée
Nourriture
Moustiques
Autre
Ne sais pas

Q9: choix multiple
Existe-t-il des moyens de ne pas attraper le paludisme ?
Moustiquaire de lit
Insecticide cutané (sur la peau)
Insecticide en bombe dans la maison ou spirale
Vêtements longs
Médicaments
Plantes
Ventilateur

Vider les coupelles d'eau
Pulvérisations dans le village (passage dans la rue)
Autre
Aucun
Ne sais pas

Q10:choix multiple

Existe-t-il des moyens de guérir du paludisme ?

Médicaments
Plantes
Autre pratique traditionnelle
Autre
Aucun
Ne sais pas

Q11: 1 choix

Si vous avez de la fièvre, ou le paludisme consultez-vous un médecin ?

Jamais
Parfois
Souvent (à chaque fois ou presque)

Q12: 1 choix (si Q11 = jamais ou parfois)

Si jamais/parfois pourquoi?

Le dispensaire est trop éloigné
La fièvre a une durée inférieure à 48h
Il n'y a pas d'autres symptômes mise à part la fièvre
Autre
Ne sais pas

Q13: 1 choix

Lieu de la dernière consultation :

CDPS de Saint Georges
Médecin libéral de Saint Georges
Cayenne
Oïapoque
Ilha Bela
Vila Brasil
Camopi
Vila Vitoria
Taparabu
Autre

Q14: 1 choix (si Q13 = oïapoque)

Si consultation à Oïapoque, où a-t-elle eu lieu ?

A l'hôpital
Chez un médecin généraliste (UBS)
Chez un médecin amérindien (CASAI)
Avec un agent de santé
Autre
Ne sais pas

Q15: 1 choix (si non passer à Q19)

Avez-vous déjà pris un médicament contre le paludisme sans ordonnance ?

Oui
Non

Q16: 1 choix

Nom du dernier médicament pris sans ordonnance :

Artecom
Coartem/Riamet
Nivaquine
Primaquine
Malarone
Lariam
Doxycycline
Aspirine
Paracétamol
Antiinflammatoire
Autre
Ne sais pas

Q17: zone de texte (si Q16 = autre)

Si autre, lequel ?

Q18: 1 choix

Lieu d'approvisionnement du médicament sans ordonnance :

CDPS de Saint Georges
Pharmacie de Saint Georges
Oïapoque
Camp d'orpaillage
Autre Guyane
Autre Brésil
Ne sais pas

Q19: 1 choix (si jamais passer à Q35)

Utilisez-vous des plantes pour vous protéger ou guérir de la fièvre ou du paludisme (malaria) ?

Jamais
Parfois
Souvent (à chaque fois ou presque)

Q21: 1 choix

Combien de plantes sont utilisées ?

1
2
3

Q22: 1 choix

N° de la plante 1 (celle qui est utilisée la plus fréquemment) :

1
2
3
4
5
6
7
8
9
10
11
12
13
14
15
16
17
18
19
20
Autre
Ne sais pas

Q23 : 1 choix (si Q22 = de 1 à 20)

Partie utilisée de la plante 1 :

Racine
Tige / Ecorce
Fleur / Fruit
Feuilles
Plante entière
Ne sais pas

Q24: 1 choix (si Q22 = de 1 à 20)

Recette de la plante 1 :

Infusion décoction dans l'eau
Macération dans l'alcool
Plante fraîche écrasée ou jus
Plante sèche broyée
Ne sais pas

Q25: 1 choix (si Q22 = de 1 à 20)

Voie d'administration (utilisation) de la plante 1 :

Orale (avalée)
Nasale (par le nez)
Application cutanée (sur la peau)
Bain
Fumigation

Autre

Q26: 1 choix (si Q21 = 2 ou 3)

N° de la plante 2

2
3
4
5
6
7
8
9
10
11
12
13
14
15
16
17
18
19
20
Autre
Ne sais pas

Q27 : 1 choix (si Q26 = de 1 à 20) et (si Q21 = 2 ou 3)

Partie utilisée de la plante 2 :

Racine
Tige / Ecorce
Fleur / Fruit
Feuilles
Plante entière
Ne sais pas

Q28: 1 choix (si Q26 = de 1 à 20) et (si Q21 = 2 ou 3)

Recette de la plante 2 :

Infusion décoction dans l'eau
Macération dans l'alcool
Plante fraîche écrasée ou jus
Plante sèche broyée
Ne sais pas

Q29: 1 choix (si Q26 = de 1 à 20) et (si Q21 = 2 ou 3)

Voie d'administration (utilisation) de la plante 2 :

Orale (avalée)
Nasale (par le nez)
Application cutanée (sur la peau)
Bain
Fumigation
Autre

Q30: 1 choix (si Q21 = 3)

N° de la plante 3 :

1
2
3
4
5
6
7
8
9
10
11
12
13
14
15
16
17
18
19
20
Autre
Ne sais pas

Q31: 1 choix (si Q30 = de 1 à 20) et (si Q21 = 3)

Partie utilisée de la plante 3 :

Racine
Tige / Ecorce
Fleur / Fruit
Feuilles
Plante entière
Ne sais pas

Q32: 1 choix (si Q30 = de 1 à 20) et (si Q21 = 3)

Recette de la plante 3 :

Infusion décoction dans l'eau
Macération dans l'alcool
Plante fraîche écrasée ou jus
Plante sèche broyée
Ne sais pas

Q33 : 1 choix (si Q30 = de 1 à 20) et (si Q21 = 3)

Voie d'administration (utilisation) de la plante 3 :

Orale (avalée)
Nasale (par le nez)
Application cutanée (sur la peau)
Bain
Fumigation
Autre

Q34: 1 choix

Comment traitez-vous votre crise de paludisme, ou votre accès de fièvre ?

J'essaie d'abord le traitement par les plantes et si cela n'est pas efficace, je prends un médicament
J'associe les plantes et les médicaments en même temps
Si le médicament n'est pas efficace j'utilise ensuite les plantes
Je n'utilise que les plantes, je ne prends pas de médicaments
Autre

Q35: 1 choix **(si non passer a Q42)**

Avez-vous déjà eu le paludisme confirmé par un test ?

Oui
Non

Q36: saisie décimale

Combien de crises de paludisme avec confirmation par un test avez-vous eu dans votre vie ?

Q38: Saisie date

Année du dernier accès palustre confirmé par un test :

Q39:1 choix

Espèce en cause pour le dernier accès confirmé par test :

Vivax
Falciparum
Autre
Ne sait pas

Q40: 1 choix

Lieu d'approvisionnement de l'antipaludique (=médicament contre la malaria) pour le dernier accès palustre confirmé par un test :

CDPS de Saint Georges
Pharmacie de Saint Georges
Oïapoque
Camp d'orpaillage
Autre Guyane
Autre Brésil

Q41: 1 choix

Avez-vous pris le traitement en totalité pour le dernier accès confirmé par un test ?

Oui
Non

Q42: 1 choix

Avez-vous des moustiquaires aux fenêtres de votre maison ?

Oui
Non

Q43: 1 choix **(si non passer à Q44 puis Q54)**

Dormez-vous sous moustiquaire ?

Oui
Non

Q44: 1 choix **(si Q43 = non)**

Si vous ne dormez pas sous moustiquaire, pourquoi ?

Cela ne protège pas des moustiques
J'aimerais mais je n'en ai pas
A cause des irritations

A cause de la toxicité
Autre

Q45: 1 choix
La moustiquaire que vous utilisez est elle impregnée d'insecticide ?
Oui
Non
Ne sais pas

Q46: 1 choix
Est-ce que votre moustiquaire a des trous ?
Oui
Non
Ne sais pas

Q47: 1 choix
Depuis quand avez-vous la moustiquaire ?
Moins de 1 an
Entre 1 et 2 ans
Entre 2 et 3 ans
Entre 3 et 4 ans
Plus de 4 ans
Ne sais pas

Q48: 1 choix
Comment avez-vous eu votre moustiquaire ?
Je l'ai achetée
On me l'a donnée

Q49: 1 choix
De quel pays vient votre moustiquaire ?
France
Brésil
Suriname
Autre
Ne sais pas

Q50: 1 choix
Comment lavez-vous votre moustiquaire ?
En machine
A la main
Je ne la lave pas

Q51: 1 choix (si Q50 = en machine ou à la main)
A quelle fréquence lavez-vous votre moustiquaire ?
Une fois par semaine
Une fois par mois
Une fois par an
Autre

Q52: 1 choix (si Q50 = en machine ou à la main)
Séchez-vous votre moustiquaire au soleil (après l'avoir lavée) ?
Oui
Non

Q53: 1 choix

Quand changez-vous votre moustiquaire ?

Quand elle est trouée
Quand il y a une distribution
Selon le mode d'emploi
Autre

Q54: 1 choix

Utilisez-vous des répulsifs individuels (en application cutanée) ?

Jamais
Parfois
Souvent (Tous les jours ou presque)

Q55: 1 choix

Utilisez-vous des insecticides pour la maison (bombe ou spirale) ?

Jamais
Parfois
Souvent (tous les jours ou presque)

Q56: 1 choix

Avez-vous des pulvérisations d'insecticide dans votre quartier ?

Jamais
Parfois (entre 1 et 3 fois par an)
Souvent (plus de 3 fois par an)
Ne sais pas

Q57: 1 choix

Pensez-vous que ces pulvérisations réduisent le nombre de moustiques ?

Oui
Non
Ne sais pas

Q58: Saisie décimale

Depuis combien de temps habitez-vous sur la commune de Saint Georges (en années) ?

Q59: choix multiple

Si vous ne dormez pas dans votre quartier habituel, ou dormez-vous ailleurs ?

Adimo
Bambou
Blondin
Bourg de Saint Georges
Esperance 1
Esperance 2
Esperance 3
Gabaret
Maripa
Onozo
Piste aviation
Savane
Tampack
Trois Paletuviers
Ouanary
Regina
Saut Maripa
Trois Sauts
Camopi
Oïapoque
Vila Brasil
Vila Vitoria
Cayenne et sa périphérie
Juminan / Taparabu
Kumaruman / Waça
Kuméné
Reste de la pointe orange
Autre Guyane
Autre Brésil

Q60: 1 choix

Etes-vous amenés à être présent / à passer sur un site d'orpaillage ?

Jamais
Parfois (moins d'une fois par mois)
Souvent (plus d'une fois par mois)

Q61: 1 choix

Des personnes travaillant sur un site d'orpaillage sont-elles amenées à être présentes dans votre maison ?

Oui
Non
Ne sais pas

Q62: 1 choix (si jamais passer à la question 67)

Allez-vous à la chasse ?

Jamais
Parfois
Souvent (plus de 3 fois par semaine)

Q63: 1 choix

A quel moment allez-vous à la chasse ?

Le jour (6h-18h)
La nuit (18h-6h)
Jour et nuit

Q64: 1 choix

Zone de chasse la plus fréquente :

1
2
3
4
5
6
7
8
9
10
Autre
Ne sais pas

Q65: 1 choix (si Q64= 1 à 10)

Zone de chasse secondaire :

1
2
3
4
5
6
7
8
9
10
Autre
Ne sais pas
Aucune

Q66: 1 choix (si Q64=1 à 10 et Q 65=1 à 10)

Zone de chasse tertiaire :

1
2
3
4
5
6
7
8
9
10
Autre
Ne sais pas
Aucune

Q67: 1 choix

Allez-vous à la pêche ?
Jamais
Parfois
Souvent (plus de 3 fois par semaine)

Q68: 1 choix
A quel moment allez-vous à la pêche ?
Jour (6h-18h)
Nuit (18h-6h)
Jour et nuit

Q69: 1 choix
Zone de pêche la plus fréquente :
1
2
3
4
5
6
7
8
9
10
Autre
Ne sais pas

Q70: 1 choix (si Q69= 1 à 10)
Zone de pêche secondaire :
1
2
3
4
5
6
7
8
9
10
Autre
Ne sais pas
Aucune

Q71: 1 choix (si Q69 = 1 à 10 et Q 70 = 1 à 10)
Zone de pêche tertiaire :
1
2
3
4
5
6
7
8
9
10

Autre
Ne sais pas
Aucune

Q72: 1 choix

Allez-vous à l'abattis ?

Jamais
Parfois
Souvent (plus de 3 fois par semaine)

Q73: 1 choix

A quel moment allez-vous à l'abattis ?

Jour (6h-18h)
Nuit (18h-6h)
Jour et nuit

Q74: 1 choix

Zone d'abattis la plus fréquente :

1
2
3
4
5
6
7
8
9
10
Autre
Ne sais pas

Q75: 1 choix (si Q 74 = 1 à 10)

Zone d'abattis secondaire :

1
2
3
4
5
6
7
8
9
10
Autre
Ne sais pas
Aucune

Q76: 1 choix (si Q74 = 1 à 10 et Q 75= 1 à 10)

Zone d'abattis tertiaire :

1
2
3
4
5
6
7
8
9
10
Autre
Ne sais pas
Aucune

Q77: 1 choix

Sexe :

Masculin
Féminin

Q78: 1 choix

Nationalité :

Français
Brésil
Suriname
Guyana
Haïtien
Chinois
Autre

Q79: 1 choix

Langue maternelle Parlée :

Français
Créole Guyanais
Créole Haïtien
Portugais du Brésil
Palikur
Patua des Karipouna
Teko
Wayampi
Kalin'ia
Sranatongo (créole du Suriname)
Saramaka
Néerlandais
Anglais du Guyana
Chinois
Hmong
Autre

Q80: 1 choix
Niveau scolaire :
N'est jamais allé à l'école
Maternelle
Primaire
Collège
Lycée
Université

Q81: 1 choix
Quelle est votre profession principale ?
Agriculteur
Chasseur
Pêcheur
Activité dans le bourg de Saint Georges
Activité dans le bourg d'Oïapoque
Piroguier
Etudiant/ à l'école
Activité a domicile (au foyer)
Travailleur-se du sexe
Activité sur un camp d'orpaillage
Retraité
Autre

Q82: saisie décimale
Combien de personnes vivent avec vous dans la maison ?

Q83: 1 choix
Quelle est votre couverture sociale ?
CMU
AME
Régime général
Pas de droits sociaux
Droits sociaux ouverts au brésil
Ne sait pas

Groupe

Q84: saisie texte
Votre PRENOM :

Q85: saisie texte
Votre NOM :

Q86: saisie date
Votre date de naissance :

Fin du groupe

---

changement1:
MERCI DE REMETTRE LA TABLETTE A L'INFIRMIER POUR LA SUITE DU QUESTIONNAIRE

Q87: saisie décimale
Température :

Q88: 1 choix
Notion de fièvre dans les 48h :
Oui
Non

Q89: saisie décimale
Poids :

Groupe

Q91s: saisie décimale
Tension systolique :

Q91d: saisie décimale
Tension diastolique :

Fin du groupe

Q92: choix multiple
Test de dépistage Rapide :
Bande C
Bande Pan
Bande Pf

Q93: saisie date
Prélèvement effectué le :

Q94: choix multiple
Prélèvement sanguin :
Tube 1 : PCR
Tube 2 : G6PD
Non réalisé

Q95: 1 choix (si Q94 = non réalisé)
Cause de non réalisation du bilan :
Impiquable
Autre

changement2:
MERCI DE REMETTRE LA TABLETTE AU MEDECIN POUR LA SUITE DU QUESTIONNAIRE

Q96: choix multiple
Antécédents / comorbidités CIM 10:
Pas d'antécédents / comorbidités
Tuberculose respiratoire avec confirmation biologique (A15)
Lèpre (A30)
Fièvre Q (A78)
Dengue (A90)
Chikungunya (A92.0)
Zika
Hépatite virale chronique (B18)
VIH (B20)
Leishmaniose (B55)
Toxoplasmose (B58)
Parasitose intestinale (B82)
Gale (B86)
Anémie par carence en fer (D50)
Drépanocytose, accès vaso-occlusif (D57.0)
Autres anémies hémolytiques héréditaires (D58)
Autres hypothyroïdies (E03)
Hyperthyroïdie (E05)
DID (E10)
DNID (E11)
Malnutrition grave (E43)
Démence sans précision (F03)
Trouble délirant persistant (F22.9)
Episode dépressif (F32)
Alcoolisme (F10.2)
Toxicomanie aux opiacés (F11.2)
Intoxication aiguë au cannabis (F12.1)
Dépendance au tabac (F17.2)
Epilepsie (G40)
Trouble de la vision sans précision (H53.9)
HTA primitive (I10)
Angine de poitrine (I20)
IDM (I21)
Cardiopathie ischémique chronique (I25)
Fibrillation et Flutter auriculaires (I48)
Embolie et thromboses artérielles (I74)
Insuffisance respiratoire Chronique (J96.1)
Ulcère gastro-jejunal (K28)
Maladie du système digestif sans précision (K92.9)
Abcès cutané - furoncle - anthrax (L02)
Insuffisance rénale chronique (N18)
Malformation congénitale et anomalie chromosomique (Q)
Saturnisme (T56.0)
Morsure ou écrasement par d'autres reptiles (W59)
Auto intoxication par des produits chimiques et substances novices (X69)

Autre antécédent / comorbidité

Q96bis: saisie texte (si Q96 = autre antécédent)
Saisir le code CIM 10 d'un autre antécédent / comorbidité associé :

Q97: 1 choix (si Q77 = F)
Grossesse en cours ?
Oui
Non

Q98: saisie décimale (si Q77=F et Q97 = oui)
Terme (en SA) :

Q99: choix multiple
Examen clinique :
Asthénie
Anorexie
Douleurs Abdominales
Diarrhées
Vomissements
Urines foncées
Céphalées
Pâleur cutanéomuqueuse
HSM (Hépatosplénomégalie)
Présence d'une splénomégalie
Examen clinique normal

Q100: saisie décimale (si Q99 = pâleur)
Valeur Hémocue

Q101: saisie décimale (si Q99 = HSM)
Flèche Hépatique (en cm) :

Q102: saisie décimale (si Q99 = splénomégalie)
Classification de Hackett :

Q104: choix multiple
Symptômes associés CIM 10:
Pas d'autre symptôme associé
Anomalie du rythme cardiaque (R00)
Souffle et autres bruits cardiaques (R01)
Toux (R05)
Dyspnée (R06.0)
Douleur thoracique sans précisions (R07.4)
Douleur abdominale et pelvienne (R10)
Nausées vomissements (R11)
Ictère (R17)
Ascite (R18)
Douleur à la miction (R30)
Hématurie (R31)
Ecoulement urétral (R36)
Coma sans précision (R40.2)
Fièvre d'origine inconnue (R50)
Céphalées (R51)
Adénopathies (R59)

Perte de poids anormale (R63.4)
Prise de poids anormale (R63.5)
Cachexie (R64)
Autre symptôme associé

Q104bis: saisie texte (si Q104 = autre symptôme)
Saisir le code CIM 10 d'un autre symptôme associé :

Q105: 1 choix
Adressé au CDPS en consultations pour motif CIM10 ?
Oui
Non

Q106: saisie décimale (si Q92 = pan ou pf)
Glycémie si TDR + :

Q107: 1 choix (si Q92 = pan ou pf)
Score de :
Glasgow
Blantyre

Q108: Saisie décimale **(si Q107 = Glasgow)**
Glasgow :

Q109: Saisie décimale (si Q107 = Blantyre)
Blantyre :

Q110: choix multiple (si Q92 = pan ou pf)
Autres signes de gravité :
Détresse respiratoire
Saignement anormal
Hémoglobinurie positive
Pas d'autre signe de gravité

Q111: 1 choix (si Q92 = pan ou pf)
Diagnostic :
Paludisme asymptomatique
Paludisme symptomatique simple
Paludisme symptomatique grave
Paludisme symptomatique compliqué

Q112: saisie texte (si Q111 = palu compliqué)
Si paludisme compliqué indiquer le motif CIM10 :

Q113: 1 choix (si Q92 = pan ou pf)
Traitement donné
Riamet
Malarone
Nivaquine
Autre

Q114: 1 choix (si Q92 = pan ou pf)
Hospitalisation ?
Oui

Non

Q115: saisie date (si Q114 = oui)
Date d'hospitalisation prévue :

Q116: saisie texte (si Q114 = oui)
Motif CIM10 d'hospitalisation :

Q117: 1 choix (si Q92 = pan ou pf)
Consultation programmée avec le généraliste ?
Oui
Non

Q118: saisie date (si Q117 = oui)
Date de consultation avec le généraliste :

Q119: 1 choix (si Q92 = pan ou pf)
Consultation programmée avec l'infectiologue des CDPS ?
Oui
Non

Q120: saisie date (si Q119 = oui)
Date de consultation avec l'infectiologue des CDPS :
