# Supplementary material for: Breaking down malaria outbreak: A multidisciplinary approach in a border village of French Guiana
Source: PLoS Negl Trop Dis. 2025 Jun 17;19(6):e0013096. doi: 10.1371/journal.pntd.0013096 (PMC12212878; doi:10.1371/journal.pntd.0013096)
Supplement: S2 File — (PDF) [file pntd.0013096.s010.pdf]

Questionnaire PALUSTOP SAISON 2(remplis par les médiateurs DAAC et l'IDE)

1) Date
Date d'aujourd'hui

2) Recherche des années dans la BD PALUSTOP 1 – saisie texte
Année de naissance du patient

3) Recherche des prénoms correspondant à l'année dans la BD PALUSTOP 1 – liste choix unique
Prénom du patient

4) Recherche des prénoms correspondant à l'année et au prenom dans la BD palustop 1 – choix unique
Nom du patient

5) Choix unique - validation du Code universel correspondant à la BD de palustop 1

6) Lecture seule
"Merci de vérifier les informations avec la personne :
Nom :
Prénom :
Année de naissance :
Code universel :

7) La personne souhaite t'elle participer à la phase 2 de PALUSTOP? (si non = fin du questionnaire)
Oui, je souhaite participer
Non, je refuse de participer
Non, La personne a déménagé
Non, nous n'avons pas retrouvé la personne

8) Choix unique
Ethnie langue maternelle 1 :
Palikur
Wayapi
Téko
Karipouna
Kalina
Galibi
Créole guyane
Créole haitien
Brésilien
Métropolitain
Saramaka
Autre

9) Choix unique
Ethnie mixte ?
Oui
Non

10) Choix unique (si Q9=oui)
Ethnie langue maternelle 2 :
Palikur
Wayapi
Téko
Karipouna
Kalina
Galibi
Créole guyane
Créole haitien
Brésilien
Métropolitain
Saramaka
Autre

11) Choix unique
Voyage dans la zone de la carbo orange l'année précédente ?
Oui
non

12) Choix multiple(si Q11=oui)
A quels endroits de la carbo orange ?
Koumene
Taparabu
Kumaruman
Manga
Autre
Ne sait pas

13) Choix unique
Heure du lever
0H (Minuit)
1H (Matin)
2H (Matin)
3H (Matin)
4H (Matin)
5H (Matin)
6H (Matin)
7H (Matin)
8H (Matin)
9H (Matin)
10H (Matin)
11H (Matin)
12H (midi)
13H (Après-midi)
14H (Après-midi)
15H (Après-midi)
16H (Après-midi)
17H (Après-midi)
18H (Après-midi)
19H (Après-midi)
20H (Après-midi)
21H (Après-midi)
22H (Après-midi)
23H (Après-midi)

14) Choix unique
Heure du coucher (idem a la liste précédente)

15) Choix unique
Etes-vous aller à l'abattis ces 6 derniers mois ?
Oui
Non

16) A quelle fréquence ? (si Q15=oui)
Au moins 3 fois par semaine ?
Au moins 1 fois par semaine ?
Au moins 1 fois par mois ?
Autre (moins de 1 fois par mois)

17) Choix unique(si Q15=oui)
En général vous partez à quelle heure à l'abattis ? (idem a la liste précédente)

18) Choix unique (si Q15=oui)
Vous arrive t'il d'y passer la nuit ?
Oui
Non

19) Choix unique(si Q15=oui)
A quelle heure revenez-vous habituellement ?(idem a la liste précédente)

20) Regardez-vous la télévision le soir ?
Oui
Non

21) Choix unique
Avez-vous eu un accès de paludisme confirmé par un test depuis l'étude palustop 1
Oui
Non

22) Choix unique (si Q21=oui)
Quel type de paludisme ?
Vivax
Falciparum
Les 2
Ne sait pas

23) Choix unique (si Q21=oui)
Qu'avez-vous fait ?
Est allé au CDPS
Est allé voir son médecin traitant
A fait de l'automédication
Autre

24) Choix unique - Affichage liste de 1 à 5 (si Q21=oui)
Nombre d'accès de paludisme depuis la phase 1 ?

25) Affichage date (si Q21=oui et Q24=1)
Date d'accès 1

26) Choix unique (si Q21=oui et Q24=1 et Q22=ne sait pas)
Quel type de paludisme pour l'accès 1

Vivax
Falciparum
Ne sait pas

27) Choix unique - (si Q21=oui et Q24=1 et (Q22=vivax ou Q26=vivax))
Avez-vous pris la Nivaquine (comprimé blanc 3 jours)?
Oui, j'ai pris le traitement complet de 3 jours
Oui, mais je n'ai pas pris la Nivaquine pendant 3 jours
Non, je n'ai pas pris de traitement
Ne sait pas

28) Choix unique - (si Q21=oui et Q24=1 et (Q22=vivax ou Q26=vivax))
Avez-vous pris la Primaquine ?
Oui, j'ai pris le traitement complet de 14 jours
Oui, mais je n'ai pas pris la Primaquine pendant 14 jours
Non, je n'ai pas pris de traitement
Ne sait pas

29) Choix unique - (si Q21=oui et Q24=1 et (Q25=falci ou Q26=falci))
Avez-vous pris le Riamet ?
Oui, j'ai pris le traitement complet de 4 jours
Oui, mais je n'ai pas pris le Riamet pendant 4 jours
Non, je n'ai pas pris de traitement
Ne sait pas

30) choix unique - (si Q21=oui et Q24=1 et (Q27=oui ou Q29=oui ou Q28=oui))
Avez-vous eu des effets secondaires ?
Oui
Non

31) choix multiple - (si Q30=oui)
Quels étaient les effets secondaires ?
Hémolyse (Pipi marron)
Fièvre
Nausées et ou vomissements
Douleurs abdominales (Mal au ventre)
Ictère (yeux jaunes)
Etourdissements
Troubles de la vision
Céphalées (mal à la tête)
Démangeaisons
Eruption cutanée
Autres

32) choix unique (si Q21=oui)
Avez-vous eu des cas de paludisme confirmé par un test dans votre maison lors de cet accès de palu dans les 2 semaines après ou avant votre accès ?
Oui
Non

33) Saisie de nombres - (si Q32=oui)
Combien ?

Les questions suivantes sont identiques à Q25 – Q33 et s'afficheront uniquement en fonction du résultat de Q24

34) Date d'accès 2
35) Quel type de paludisme pour l'accès 2
36) Avez-vous pris la Nivaquine (comprimé blanc 3 jours)?
37) Avez-vous pris la Primaquine ?
38) Avez-vous pris le Riamet ?
39) Avez-vous eu des effets secondaires ?
40) Quels étaient les effets secondaires ?
41) Avez-vous eu des cas de paludisme confirmé par un test dans votre maison lors de cet accès de palu dans les 2 semaines après ou avant votre accès ?
42) Combien ?

43) Date d'accès 3
44) Quel type de paludisme pour l'accès 3
45) Avez-vous pris la Nivaquine (comprimé blanc 3 jours)?
46) Avez-vous pris la Primaquine ?
47) Avez-vous pris le Riamet ?
48) Avez-vous eu des effets secondaires ?
49) Quels étaient les effets secondaires ?
50) Avez-vous eu des cas de paludisme confirmé par un test dans votre maison lors de cet accès de palu dans les 2 semaines après ou avant votre accès ?
51) Combien ?

52) Date d'accès 4
53) Quel type de paludisme pour l'accès 4
54) Avez-vous pris la Nivaquine (comprimé blanc 3 jours)?
55) Avez-vous pris la Primaquine ?
56) Avez-vous pris le Riamet ?
57) Avez-vous eu des effets secondaires ?
58) Quels étaient les effets secondaires ?
59) Avez-vous eu des cas de paludisme confirmé par un test dans votre maison lors de cet accès de palu dans les 2 semaines après ou avant votre accès ?
60) Combien ?

61) Date d'accès 5
62) Quel type de paludisme pour l'accès 5
63) Avez-vous pris la Nivaquine (comprimé blanc 3 jours)?
64) Avez-vous pris la Primaquine ?
65) Avez-vous pris le Riamet ?
66) Avez-vous eu des effets secondaires ?
67) Quels étaient les effets secondaires ?
68) Avez-vous eu des cas de paludisme confirmé par un test dans votre maison lors de cet accès de palu dans les 2 semaines après ou avant votre accès ?
69) Combien ?

70) Choix unique

Avez-vous eu un épisode de diarrhée de plus de 3 jours depuis PALUSTOP 1 ?

Oui

Non

71) Choix unique

Avez-vous eu un épisode de fièvre (>38C°) depuis PALUSTOP 1 ?

Oui

Non

72) Saisie nombre(si Q71=oui)

Combien de fois avez-vous eu de la fièvre depuis l'année dernière?

73) Saisie nombre (si Q71=oui)
Et combien de test du paludisme avez-vous fait en cas de fièvre ?

74) Lecture seule
MERCI D'ENREGISTRER ET DE REMETTRE LA TABLETTE A L'INFIRMIER POUR LA SUITE DU QUESTIONNAIRE

75) Choix unique
Notion de fièvre dans les 48h
Oui
Non

76) Règle graduée
Température

77) Choix multiple - (Si Q75=oui ou si Q76>=38)
Test de dépistage Rapide
Bande C
Bande Pan
Bande Pf
Non fait

78) Date et heure
Prélèvementeffectué le :

79) Choix unique
Prélèvement sanguin
Tube PCR
Non réalisé

80) Choix unique
Cause de non réalisation du bilan
Impiquable
Autre
