# Supplementary material for: Breaking down malaria outbreak: A multidisciplinary approach in a border village of French Guiana
Source: PLoS Negl Trop Dis. 2025 Jun 17;19(6):e0013096. doi: 10.1371/journal.pntd.0013096 (PMC12212878; doi:10.1371/journal.pntd.0013096)
Supplement: S4 File — (PDF) [file pntd.0013096.s012.pdf]

“Breaking down a malaria outbreak”: Illustration of the context of the multidisciplinary investigation in the border village of Trois-Palétuviers in French Guiana in 2017 and 2018.

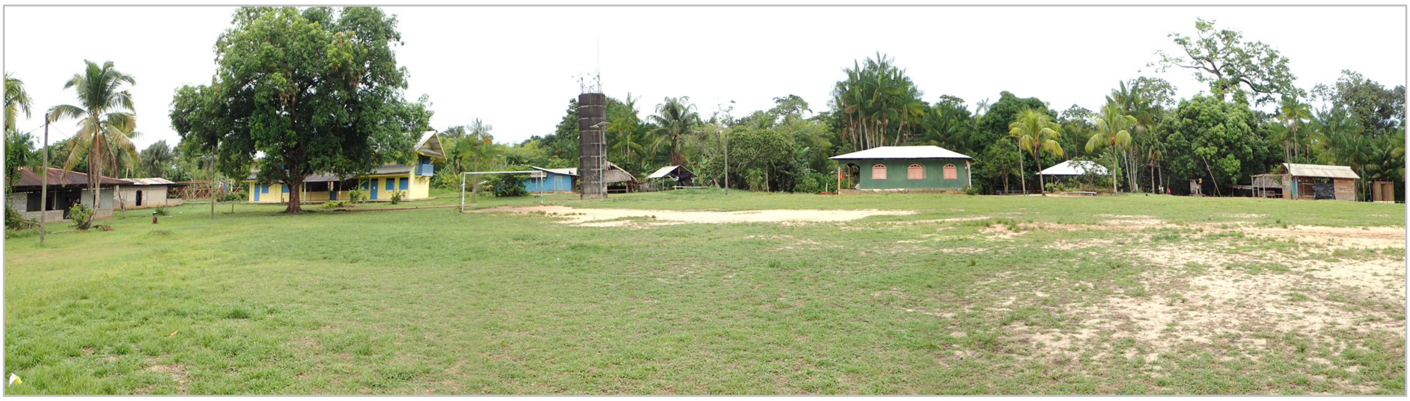

Figure C1. Trois-Palétuviers is located along the Oyapock river, at 1-hour boat trip from the city of Saint-Georges de l’Oyapock, surrounded by the Amazonian forest. This isolated situation restricted access to healthcare and the number of mosquito traps deployed during the study (photo credit: Guillaume Lacour).

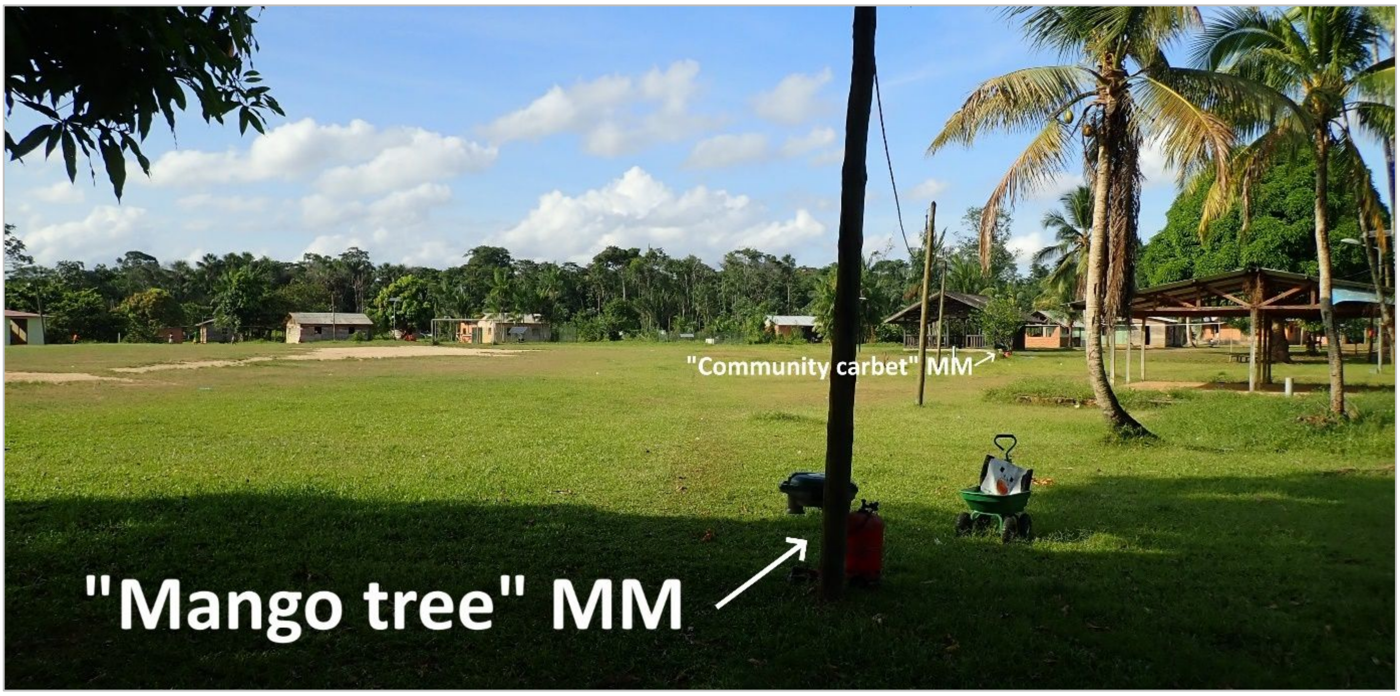

Figure C2. Mosquito magnet traps installed near the old Mango tree and the community carbet. Only Mango trees and coconut trees grow inside the Trois-Palétuviers village. Sandy place to play football is visible at the lefty of the picture (photo credit: Guillaume Lacour).

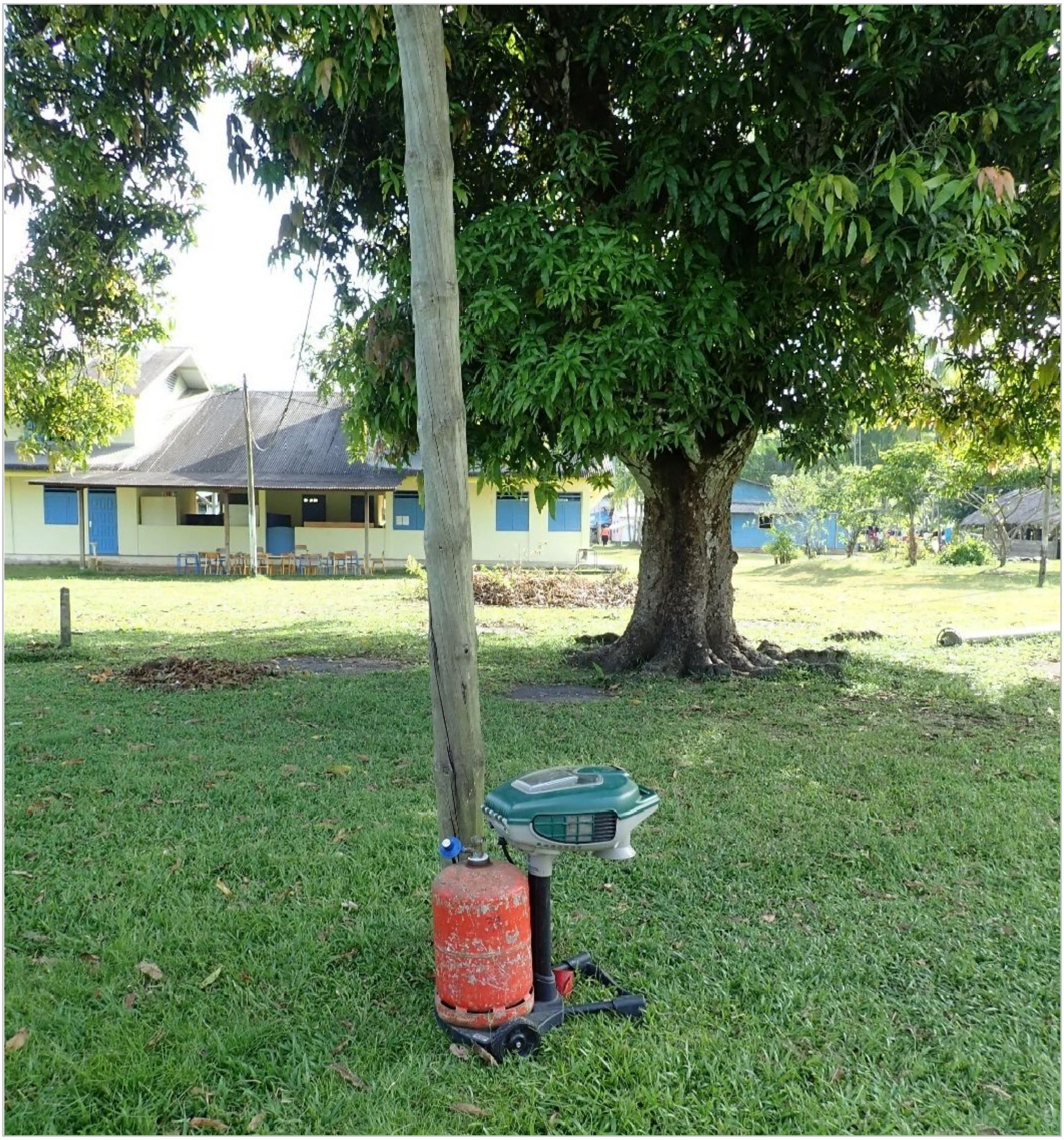

Figure C3. Mosquito magnet trap in front of the old Mango tree. Mango trees have a large tree crown, providing shade during the day (rare in the village, outside of homes) and nectar to insects. Mango trees are potential resting sites for mosquitoes, without known preferences for *Anopheles darlingi*. The school is visible behind the Mango tree (photo credit: Guillaume Lacour).

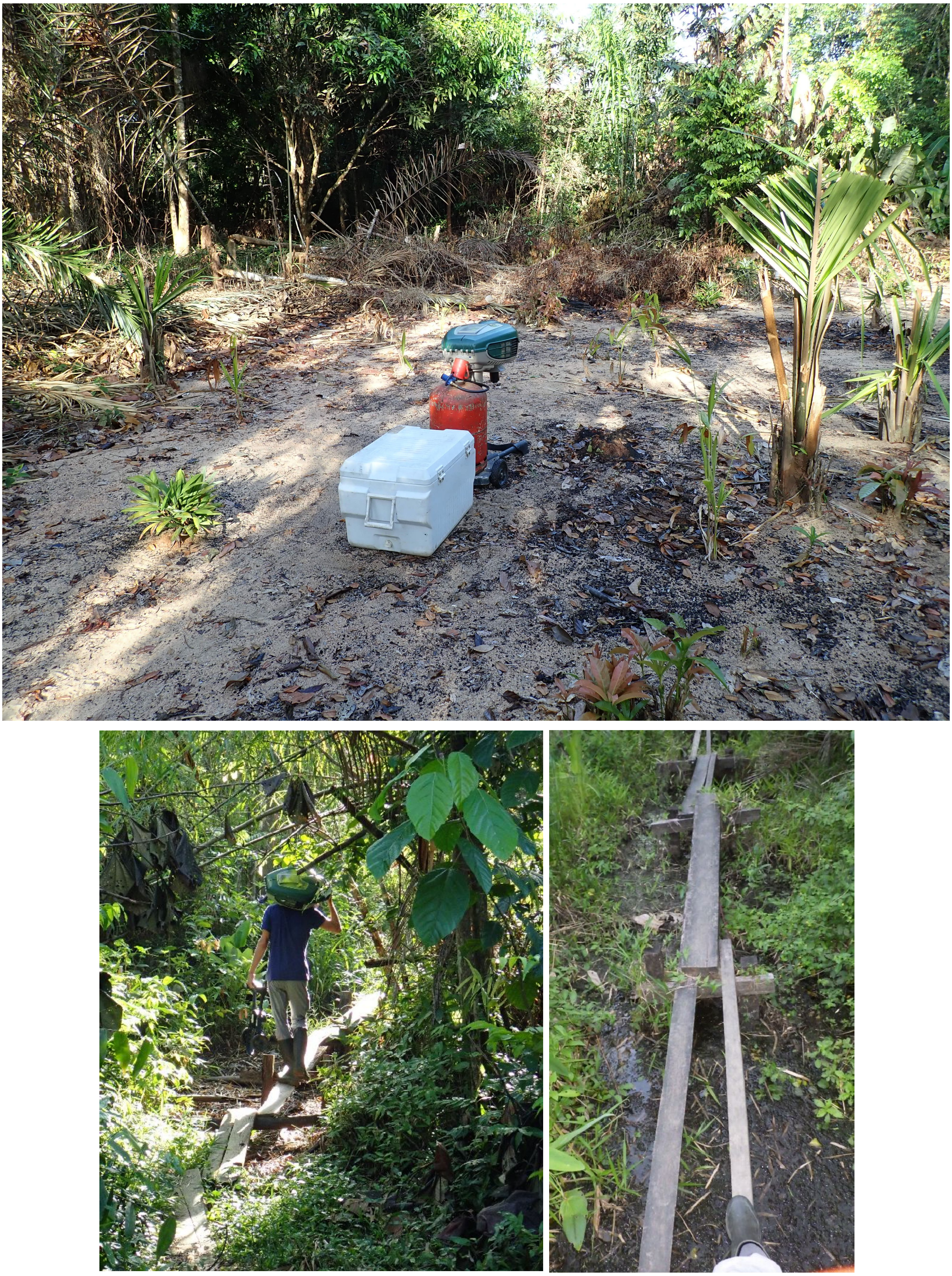

Figure C4. Slash-and-burn field where a Mosquito Magnet trap was installed. The path from the village to the fields was marshy, requiring a second team to manage only the trap deployed in the slash-and-burn field during the night (photo credit: Guillaume Lacour).
